# Systemic immunometabolic profiling classifies cisplatin sensitivity states using interpretable machine learning

**DOI:** 10.1016/j.isci.2026.115037

**Published:** 2026-02-17

**Authors:** Emily Y. Kim, Diane C. Lim, Yujie Wang, Edison Q. Kim, Chunjing Wu, Ankita Paul, Cheng-Bang Chen, Medhi Wangpaichitr

**Affiliations:** 1 Research Services, Miami VA Healthcare System, Miami, FL 33125, USA; 2 South Florida Veterans Affairs Foundation for Research and Education, Inc., Miami, FL 33125, USA; 3 Ellen and Ronald Caplan Cancer Center, The Wistar Institute, Philadelphia, PA 19104, USA; 4 Department of Cancer Biology, School of Health Professions and Sciences, Philadelphia College of Osteopathic Medicine, Philadelphia, PA 19131, USA; 5 Department of Medicine, Miami VA Medical Center, Miami, FL 33125, USA; 6 Department of Medicine, Miller School of Medicine, University of Miami, Miami, FL 33136, USA; 7 Department of Industrial and Systems Engineering, University of Miami, Coral Gables, FL 33146, USA; 8 Department of Electrical and Computer Engineering, Drexel University, Philadelphia, FL 19104, USA; 9 Department of Surgery, Cardiothoracic Surgery, University of Miami, Miami, FL 33136, USA

**Keywords:** Metabolomics, Computing methodology, Machine learning

## Abstract

Cisplatin resistance limits the effectiveness of platinum-based chemotherapy for lung adenocarcinoma, yet practical systemic diagnostics for cisplatin sensitivity are lacking. We developed ImmunoMetabolic Profiling Analysis and Classification Tool (IMPACT), an interpretable machine learning pipeline that selects the best performing model and reduces it to a minimal, mechanistically informative feature set via recursive feature elimination. In a syngeneic orthotopic model, we quantified 25 serum amino acids and 16 immune cell populations across bone marrow, spleen, lung, and mediastinal lymph nodes to capture systemic immunometabolic states. IMPACT classified cisplatin-sensitive versus cisplatin-resistant tumors with high accuracy (AUC = 0.950), driven primarily by bone marrow MDSCs and serum glutamine. Using the same framework, we also classified Cancer (CS + CR) versus no cancer controls with high accuracy (AUC = 0.955), with lung MDSCs and phosphoserine among the top features.

## Highlights

• Interpretable machine learning reveals coordinated metabolic and immune interactions
• Systemic immunometabolic profiling classifies cisplatin-sensitive vs. resistant tumors
• Systemic immunometabolic profiling classifies cancer from noncancer states

## Graphical abstract

**Figure:**
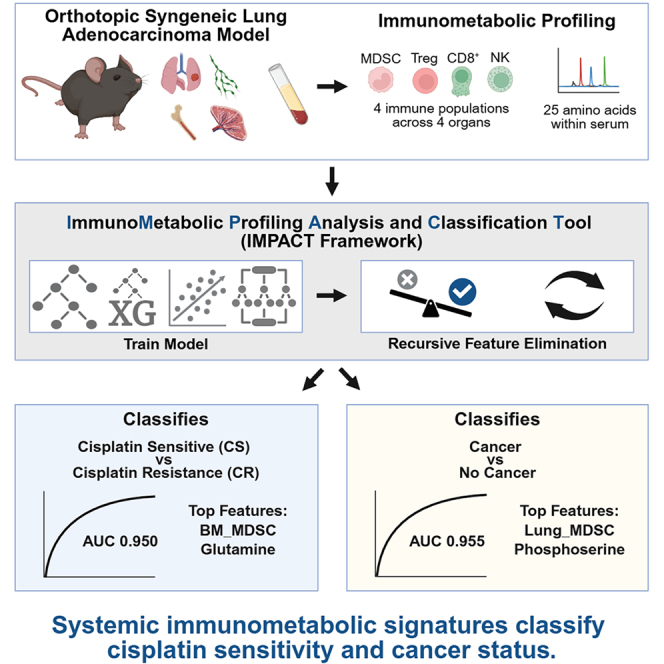

## Introduction

Platinum-based chemotherapies, including cisplatin, remain standard first-line treatments for non-small cell lung cancer (NSCLC). Although initially effective, most patients ultimately develop resistance,^1^^,^^2^ limiting meaningful long-term benefit. Despite extensive research revealing diverse biological mechanisms underlying platinum resistance, there are still no clinically available diagnostic tests that can identify cisplatin sensitivity before or after treatment begins. A reliable, biologically grounded diagnostic tool would help clinicians determine whether to initiate or modify its use proactively, rather than after resistance has already manifested as accelerated tumor progression.

Developing such a diagnostic test is challenging because cancer is inherently heterogeneous and continuously evolving.^3^^,^^4^ In addition, intrinsic tumor mechanisms alone cannot fully account for variability in treatment outcomes. Growing evidence shows that system-level immunometabolic interactions, spanning circulating amino acid availability and multiorgan expansion of myeloid-derived suppressor cells (MDSCs) and regulatory T cells (Tregs), shape tumor metabolism and the tumor immune microenvironment, contributing to immunosuppression,^5^ progression, and treatment resistance. Thus, effective diagnostic strategies must capture these integrated, multiorgan relationships, rather than focus on isolated mechanisms.^6^ This system-level immunometabolic profiling is recognized to be important but remains unfulfilled.

In parallel, several complementary biomarker strategies have been developed for lung adenocarcinoma/NSCLC that primarily interrogate tumor-intrinsic programs and/or the tumor microenvironment (TME). Computational toolkits such as Immuno-Oncology Biological Research enable standardized multi-omics interrogation of TME composition and tumor-immune interactions.^7^^,^^8^ Epigenetic signatures, including methylation-based prognostic models, provide another route to capture relatively stable tumor heterogeneity and stratify outcomes.^9^ Transcriptomic gene signature approaches integrating multiple regulatory molecules have also been proposed to link TME organization to prognosis and immunotherapy relevance.^10^ These tumor/TME-centric strategies are complementary to systemic immunometabolic profiling, which emphasizes host-level immune metabolic coupling across compartments and may provide an orthogonal, peripherally accessible layer of information for translation.

Recent advances in big data analytics and machine learning (ML) offer a practical way to analyze complex, high-dimensional biological datasets that emerge from system-level immunometabolism.^6^ When applied rigorously, ML can detect consistent patterns, reduce feature redundancy, and reveal physiologically interpretable structures even when sample sizes are constrained, an unavoidable reality in both preclinical and clinical cancer studies. Importantly, interpretable ML methods such as recursive feature elimination (RFE) do not replace biological insight; rather, they organize it by identifying the most informative markers within a larger physiological network. This allows ML models to highlight biologically meaningful signatures of resistance early, independent of any single resistance mechanism.

To address these needs, we developed IMPACT, the immunometabolic profiling analysis and classification tool, specifically to be interpretable and reproducible. Rather than rely on a single algorithm or opaque metrics, we required consistency across four distinct supervised models and stability of feature rankings under recursive elimination. This approach ensures that IMPACT highlights immunometabolic features that are repeatedly identified across independent computational strategies, reducing the risk of overfitting and increasing confidence that these signals reflect real physiological patterns rather than algorithmic artifacts.

IMPACT integrates 41 immunometabolic features per mouse (25 serum amino acids; 16 immune cell populations across lung, spleen, mediastinal lymph node, and bone marrow) and employs a two-step workflow. Step 1 trains supervised models to rank immunometabolic features most associated with cisplatin-sensitive (CS) versus cisplatin-resistant (CR) states; Step 2 applies RFE to derive a reduced, stable set of discriminators. We then extended the same framework to distinguish cancer from noncancer states. Because immunometabolic remodeling spans multiple organs and metabolic axes, we reasoned that systemic features would encode reproducible patterns reflecting immune suppression, metabolic reprogramming, and tumor presence.

This study represents a step toward developing a minimally invasive, biologically interpretable, ML-driven framework for classifying cisplatin sensitivity states and cancer presence. By grounding our approach in systemic immunometabolic profiling and validating it across multiple algorithms, our goal is to provide a platform that is both scientifically rigorous and accessible to translational researchers and clinicians.

## Results

### Cisplatin sensitivity has a systemic immunometabolic signature dominated by bone marrow_myeloid-derived suppressor cells and glutamine

#### Step 1: Initial and full model training yields a stable model

**Figure 1:**
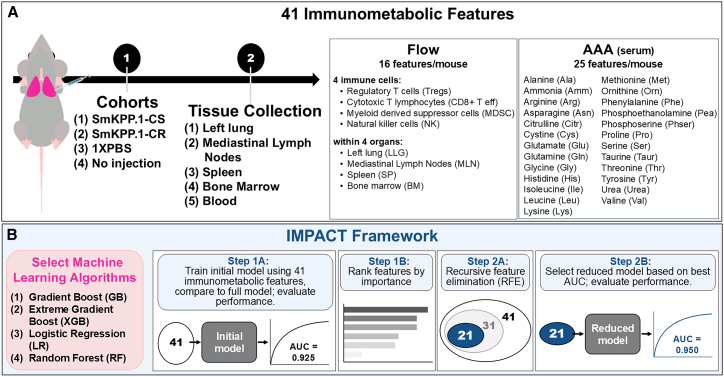
Study design and overview of the IMPACT framework (A) Immunometabolic dataset generation. Four mouse cohorts are analyzed: (1) cisplatin-sensitive tumors (SmKPP.1-CS), (2) cisplatin-resistant tumors (SmKPP.1-CR), (3) 1XPBS injection controls, and (4) no injection controls. A total of 41 immunometabolic features are quantified, including 16 flow cytometry-derived immune cell populations (Tregs, CD8^+^ effector T cells (CD8^+^ T eff), MDSCs, and NK cells across left lung (LLG), mediastinal lymph node (MLN), spleen (SP), and bone marrow (BM)) and 25 serum amino acids. (B) IMPACT workflow. Step 1: Train machine learning models (we selected Gradient Boosting (GB), Extreme Gradient Boosting (XGB), Logistic Regression (LR), and Random Forest (RF)) on the 41-feature dataset and rank features by model-derived importance. Step 2: Apply recursive feature elimination (RFE) to iteratively remove the lowest importance feature, then retrain the model, resulting in an unbiased, interpretable, dimension-reduced feature list.

To evaluate whether systemic immunometabolic signals encode cisplatin sensitivity, we trained IMPACT on 41 features from Cohort 1, mice implanted orthotopically (left lung) with CS lung adenocarcinoma cells, and Cohort 2, mice implanted with CR lung adenocarcinoma cells (Figure 1A). These features included 25 serum amino acids and 16 immune cell populations across the left lung (LLG), mediastinal lymph node (MLN), spleen (SP), and bone marrow (BM). We developed IMPACT to compare four machine learning algorithms (Figure 1B) to allow transparent reproducibility.

In the initial model (Table S1), each of the four algorithms, Gradient Boost (GB), XGBoost (XGB), Logistic Regression (LR), and Random Forest (RF), performed above baseline discriminatory thresholds. RF returned the strongest performance (AUC 0.925 ± 0.058; Accuracy 0.854 ± 0.081; Sensitivity 0.865 ± 0.108; Specificity 0.844 ± 0.097; Precision 0.851 ± 0.083; F1 score 0.854 ± 0.081). GB, XGB, and LR returned mean AUCs of 0.889 ± 0.069, 0.876 ± 0.063, and 0.705 ± 0.085, respectively. When we added 3 covariates, age of mouse, gender and time elapsed from cancer initiation to harvest, to the 41 immunometabolic features (full model; Table S2), RF again performed best (AUC 0.953 ± 0.040; Accuracy 0.880 ± 0.070; Sensitivity 0.898 ± 0.089; Specificity 0.863 ± 0.099; Precision 0.872 ± 0.079; F1 score 0.882 ± 0.069). GB, XGB, and LR returned mean AUCs of 0.906 (±0.049), 0.921 (±0.047), and 0.723 (±0.086), respectively. DeLong comparisons between initial and full models (Table S3) provided evidence that these covariates did not impact model performance. Therefore, we did not add the covariates to Step 2. All performance metrics are reported as mean ± SD across 30 repeated stratified 80/20 train-test splits. For threshold-dependent metrics (accuracy, sensitivity, specificity, precision, F1), the operating threshold was selected for each model by maximizing F1 on pooled held-out predictions across all resamples and then applied to each resample to summarize performance.

#### Step 2: Recursive feature elimination identifies bone marrow_myeloid-derived suppressor cells and glutamine as consistent top-ranked features discriminating cisplatin-sensitive vs. cisplatin-resistant across algorithms

**Figure 2:**
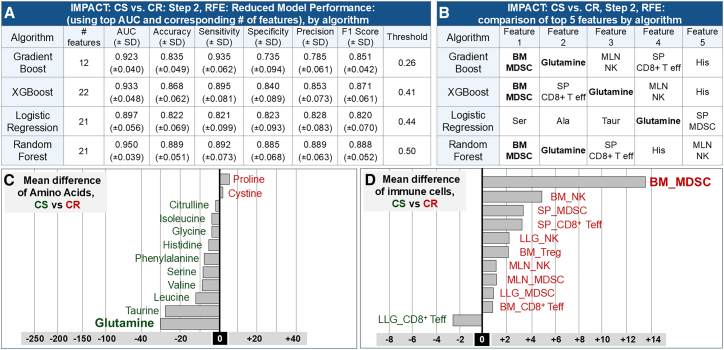
Immunometabolic classification of cisplatin-sensitive and cisplatin-resistant tumors (A) Algorithm performance. Comparison of AUC, accuracy, sensitivity, and specificity across four supervised algorithms distinguishing cisplatin-sensitive (CS) from cisplatin-resistant (CR) tumors. Random Forest using the reduced feature set returned the highest overall performance (AUC 0.950 ± 0.039; Table S8). (B) Top-ranked discriminators. Recursive feature elimination (RFE) identified the five most informative features for each algorithm. BM_MDSC and serum glutamine are repeatedly selected among the top predictors across models. (C) Directional differences in serum amino acids (CS vs. CR). Values represent mean differences (μmol/L); negative values (green) indicate higher abundance in CS mice and positive values (red) indicate higher abundance in CR mice. CS mice showed higher glutamine and other metabolites associated with anabolic/glycolytic programs (e.g., taurine, serine, and leucine), whereas CR mice showed higher proline and cystine, consistent with divergent glycolytic versus oxidative systemic states. (D) Directional differences in immune cell populations (CS vs. CR)*.* Values represent mean differences in immune subset abundance (percentage of positive cells per 10,000 counted); negative values (green) indicate higher abundance in CS mice and positive values (red) indicate higher abundance in CR mice. CR tumors are associated with expanded BM_ and SP_MDSCs and increased NK populations, while CS tumors showed higher LLG_CD8^+^ Teff frequencies, consistent with effector-permissive immunity versus myeloid-driven suppression.

To derive a compact, interpretable feature set, we next applied RFE. First, we generated a 41-feature importance rankings list for all algorithms using the caret Varlmp metric. Next, we removed the lowest-ranked feature, then retrained the model until one feature remained; for each iteration (removing the lowest-ranked feature), we provide the mean AUC (Tables S4–S8). The iteration with the highest AUC was selected as the reduced model (Figure 2A). RF again returned the strongest performance (AUC 0.950 ± 0.039; Accuracy 0.889 ± 0.051; Sensitivity 0.892 ± 0.073; Specificity 0.885 ± 0.068; Precision 0.889 ± 0.063; F1 score 0.888 ± 0.052). GB, XGB, and LR returned mean AUCs of 0.923 (±0.040), 0.933 (±0.048), and 0.897 (±0.056), respectively. Comparing the RF initial model (AUC 0.925 ± 0.058; Table S1) and covariate full model (AUC 0.953 ± 0.040; Table S2), the reduced model (AUC 0.950 ± 0.039) was effectively indistinguishable, confirming that a compact feature set retains most of the predictive power. Notably, key discriminators (e.g., BM_MDSC and glutamine) recur across multiple nonlinear algorithms, providing an internal robustness check that performance is not driven by a single model class.

The top five features for GB, XGB, LR, and RF are shown in Figure 2B. BM_MDSC and glutamine appeared among the top-ranked features across GB, XGB, and RF models, with SP_CD8^+^ Teff and histidine also consistently prioritized by the nonlinear algorithms. To corroborate the machine learning approach and provide directional interpretation, Figure 2C shows mean differences of amino acids (μmol/L) and Figure 2D shows mean differences of immune cell populations (percentage of positive cells per 10,000 counted). Negative values (green) reflect higher abundance in CS mice; positive values (red) reflect higher abundance in CR mice. CS mice displayed greater glutamine, taurine, histidine, and LLG_CD8^+^ Teff. CR mice displayed higher proline, cystine, and BM_MDSC.

### Cancer presence has a systemic immunometabolic signature dominated by left lung_myeloid-derived suppressor cells and phosphoserine

To assess IMPACT’s generalizability, we extended the same framework to distinguish cancer (CS and CR combined) from noncancer states. Before model training, we verified that injecting the lung did not contribute to group differences. Cohort 3 (1XPBS injection) and Cohort 4 (no injection) did not differ significantly across 41 features (Table S9), supporting their use as a combined “*No Cancer*” group.

#### Step 1: Initial and full model training yields a stable model

The initial model for cancer detection (Table S10) again showed that all algorithms performed above baseline discriminatory thresholds. RF produced the strongest performance (AUC 0.942 ± 0.047; Accuracy 0.901 ± 0.037; Sensitivity 0.965 ± 0.034; Specificity 0.715 ± 0.126; Precision 0.909 ± 0.036; F1 score 0.935 ± 0.023). GB, XGB, and LR returned mean AUCs of 0.938 ± 0.045, 0.923 ± 0.055, and 0.783 ± 0.110, respectively.

When the same 3 covariates were added for the full model (Table S11), RF again performed best (AUC 0.967 ± 0.040; Accuracy 0.929 ± 0.031; Sensitivity 0.988 ± 0.021; Specificity 0.761 ± 0.125; Precision 0.925 ± 0.037; F1 score 0.954 ± 0.020). GB, XGB, and LR returned mean AUCs of 0.966 (±0.036), 0.968 (±0.033), and 0.805 (±0.068), respectively. As in the *CS* vs*. CR* analysis, the difference between initial and full models for *cancer* vs. no cancer using a DeLong test (Table S12) was not statistically significant, indicating that covariate inclusion did not significantly change model discrimination. Therefore, we did not add the covariates to Step 2.

#### Step 2: Recursive feature elimination identifies left lung_myeloid-derived suppressor cells and phosphoserine as consistent top-ranked features discriminating cancer vs. No cancer across algorithms

**Figure 3:**
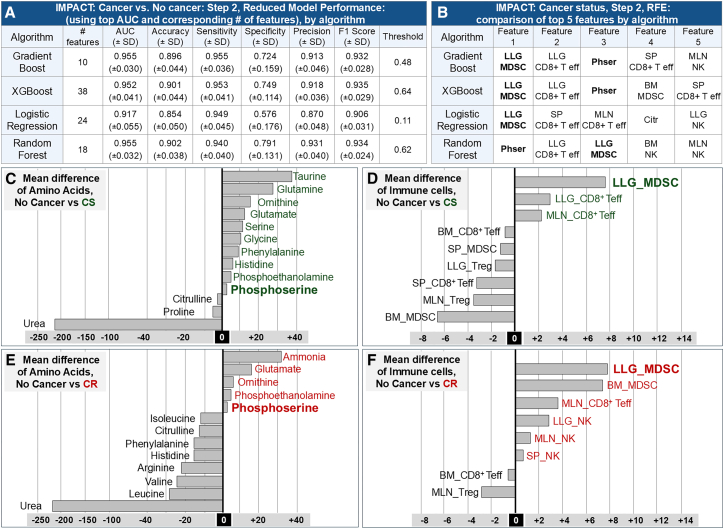
Applying IMPACT to cancer detection, related to Figure S2 (A) Model performance for cancer vs. no cancer classification. CS and CR samples are merged as the “Cancer” group and compared with uninjected and 1XPBS controls. Random Forest again showed the strongest performance (AUC 0.955 ± 0.032; Table S17). (B) Top-ranked discriminators. Recursive feature elimination identified LLG_MDSC as the dominant cancer-associated feature across all algorithms, with phosphoserine also consistently prioritized, highlighting early myeloid remodeling and altered serine/one-carbon-linked metabolism as systemic hallmarks of malignancy. (C and D) Mean differences in serum amino acids and immune cell populations (CS vs. no cancer). Values represent mean differences; negative values (black) indicate higher abundance in no cancer controls, and positive values (green) indicate higher abundance in CS mice. Relative to controls, CS mice displayed increases in taurine and phosphoserine and higher LLG_CD8^+^ Teff, along with reduced urea and elevated MDSCs in specific organs. (E and F) Mean differences in serum amino acids and immune cell populations (CR vs. no cancer). Values represent mean differences; negative values (black) indicate higher abundance in *No Cancer* controls, and positive values (red) indicate higher abundance in CR mice. CR mice exhibited pronounced phosphoserine elevation, strong urea depletion, and increased MDSC and NK cell abundance, demonstrating that cancer presence, independent of cisplatin sensitivity, induces a consistent immunometabolic signature dominated by nitrogen/one-carbon metabolism remodeling and myeloid reprogramming.

We repeated RFE, generating a 41-feature importance rankings list for all algorithms, removing the lowest-ranked feature, then retrained each model, providing the mean AUC for each iteration (Tables S13–S17). The iteration with the highest AUC was selected as the reduced model (Figure 3A).

**Figure 4:**
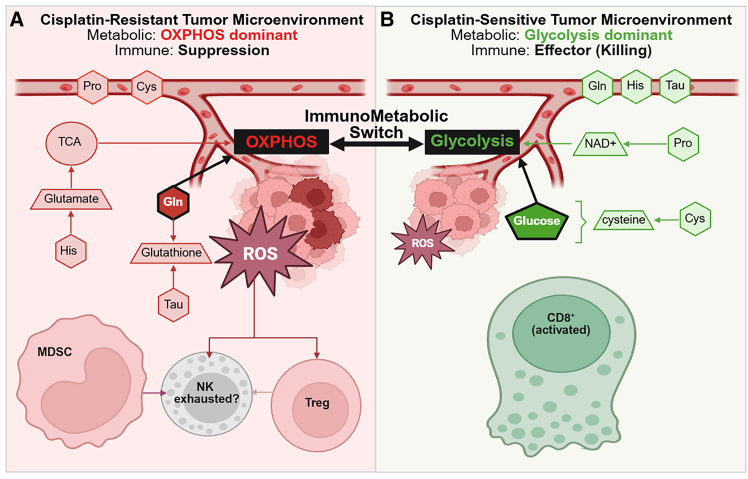
Systemic immunometabolic model of cisplatin sensitivity and resistance derived from IMPACT Conceptual schematic integrating the most stable systemic discriminators of cisplatin-sensitive (CS) versus cisplatin-resistant (CR) tumors identified by IMPACT with an evidence-based model of glycolysis-to-OXPHOS immunometabolic switching. Across algorithms and recursive feature elimination, stability analysis converged on a compact *CS* vs*. CR* signature comprising BM_MDSC, SP_CD8^+^ Teff, and circulating glutamine, histidine, and proline, together with concordant directional trends in serum amino acids and multi-organ immune subsets (Figures 2B–2D, Tables S4–S7). Substrates enclosed by the dotted boundary are not measured in this study but will be assessed in future work. (A) CR state: OXPHOS-dominant, ROS-buffering, and myeloid-skewed systemic signature. Relative to CS, CR mice exhibit higher serum proline and cystine, increased MDSC abundance (particularly in bone marrow and spleen), and expanded NK-cell populations (Figures 2C and 2D). These measured systemic associations are consistent with a more oxidative/ROS-buffering immunometabolic program linked to reduced cisplatin response. (B) CS state: Glycolysis-dominant and effector-permissive systemic signature. CS mice show higher circulating glutamine, histidine, and taurine (along with other metabolites associated with anabolic/glycolytic programs, e.g., serine and leucine) and higher lung CD8^+^ effector T cell frequencies (LLG_CD8^+^ Teff) (Figures 2C and 2D), consistent with an effector-permissive systemic immune state. Interpretation note. Amino acids are quantified in serum; therefore, relative increases reflect altered systemic availability (e.g., increased production/release or reduced utilization), whereas relative decreases may reflect increased utilization/uptake. This schematic summarizes measured systemic patterns and hypothesized pathways and does not directly quantify intratumoral metabolism or immune-cell functional state.

The top five discriminating features for each model are summarized in Figure 3B. LLG_MDSC and phosphoserine appeared among the top-ranked features across all four models, with LLG_CD8^+^ Teff and SP_CD8^+^ Teff also consistently prioritized by the nonlinear algorithms. To corroborate the machine learning approach and provide directional interpretation, we show mean differences of amino acids and immune cells between no cancer vs. CS (Figures 3C and 3E) and no cancer vs. CR (Figures 3D and 3F). Negative values (blue) reflect higher abundance in no cancer mice; positive values reflect higher abundance in CS (green) or CR (red) mice. CS mice displayed increased taurine, glutamine, serine, histidine, LLG_MDSC, LLG_CD8^+^ Teff, and MLN_CD8^+^ Teff relative to no cancer. CR mice displayed increased ammonia, glutamate, phosphoserine, LLG_MDSC, BM_MDSC, and MLN_CD8^+^ Teff.

### Core immunometabolic axes remain stable across different classification tasks and across different models

Across both classification tasks, *CS* vs*. CR* and *cancer* vs. no cancer, reduced feature models closely matched or exceeded initial/full model performance, demonstrating that discrimination arises from core systemic immunometabolic signals, not model dimensionality. Feature stability, consistent rankings across algorithms, and concordant directional trends in amino acids and immune subsets all support a unified systemic landscape that differentiates cisplatin sensitivity and cancer presence. Together, these results highlight the robustness, interpretability, and biological coherence of the IMPACT framework.

Feasibility note for future translation: In this discovery dataset, IMPACT prioritized a compact set of high-value discriminators spanning both blood accessible serum metabolites and organ-resolved immune features. While multiorgan immune profiling (including bone marrow) is not directly clinically feasible and was used here to localize mechanistic anchors of systemic remodeling, the consistent prioritization of serum metabolites (e.g., glutamine for CS vs. CR; phosphoserine for cancer vs. no cancer) motivates future development of serum and peripheral blood-based reduced classifiers. Such reduced panels (e.g., targeted blood immune phenotypes paired with serum amino acids) could be trained and validated in independent cohorts to benchmark how much discriminatory signal can be recovered without invasive tissue sampling, and to define the minimal feature set needed for robust clinical performance.

## Discussion

In this study, we introduce IMPACT, an interpretable two-step machine learning framework that integrates targeted serum amino acid profiling with multiorgan immune phenotyping to map systemic immunometabolic states. By combining cross-algorithm model training with RFE, IMPACT classified both cisplatin sensitivity (*CS* vs. *CR*) and cancer status (*cancer* vs. *no cancer*) with high accuracy while converging on compact, biologically coherent feature signatures. Together, these results support the concept that therapy response and malignancy are reflected not only within the tumor microenvironment but also as a coordinated systemic immunometabolic phenotype accessible from peripheral sampling.

Because these signatures were discovered in a syngeneic mouse model, we interpret them as a discovery-stage systems *map* rather than a clinically deployable assay. In this context, bone marrow immune features serve as mechanistic anchors for discovery; a clinically feasible implementation would require (i) assay redesign toward clinically tractable, minimally invasive readouts (peripheral blood immune phenotypes and serum metabolites), (ii) training and validation in independent human lung adenocarcinoma/NSCLC cohorts with standardized pre-analytic handling, and (iii) explicit control for host covariates (comorbidities, medications, treatment history, and nutritional state). Importantly, human evidence supports the relevance of circulating MDSC populations in NSCLC: peripheral blood MDSCs have been reported to predict recurrence after surgery^11^ and meta-analytic evidence links higher circulating MDSCs to worse outcomes across cohorts.^12^ In addition, glutamine-centered immunometabolic programs regulate suppressive myeloid biology in human cancer^10^ and therapeutic targeting of glutamine metabolism can remodel suppressive myeloid compartments and enhance antitumor immunity,^13^ supporting the biological plausibility of the top-ranked MDSC and glutamine features highlighted by IMPACT in distinguishing CS from CR.

Our systemic, organism-level perspective is intended to complement, not replace, tumor-centric prognostic models and therapeutic sensitization strategies. For example, tumor intrinsic transcriptional regulatory programs (including microRNA-linked pathways) can shape drug sensitivity in NSCLC and provide mechanistic entry points for targeted sensitization.^14^ Similarly, strategies aimed at reprogramming immunosuppression and increasing tumor immunogenicity, including nanomedicine-enabled approaches that boost immunogenic cell death, highlight additional routes to improve treatment responsiveness and augment immunotherapy efficacy.^15^ In future studies, integrating systemic immunometabolic readouts (IMPACTs) with tumor/TME multiomics models could support a more complete, multiscale map of resistance biology and help prioritize rational combination or monitoring strategies.

Although IMPACT is intentionally centered on systemic immunometabolic features, tumor intrinsic molecular programs, including transcriptomic gene expression signatures and post-transcriptional regulation, remain important determinants of prognosis and therapeutic response in lung adenocarcinoma/NSCLC. For example, integrated multigene signatures have been reported to associate with lung adenocarcinoma prognosis and tumor immune cell infiltration and to inform immunotherapy-oriented stratification.^10^ We therefore view IMPACT as a complementary, blood accessible host state layer that can be integrated with tumor-derived transcriptomic, epigenetic, and post-transcriptional markers in future multiomic models, with the goal of improving prediction while directly linking systemic physiology to tumor intrinsic resistance biology.

### The immunometabolic switch: *CS* vs. *CR*

Our results support the idea that cisplatin sensitivity and resistance are reflected in a coordinated, system-level “immunometabolic switch” that is recoverable from peripheral amino acid profiling together with multiorgan immune composition (Figure 2, Tables S1–S8). In this framework, the CS state aligns with an effector-permissive immune landscape (including higher lung CD8^+^ effector T cell signals) coupled to a glycolysis-dominant metabolic program that is commonly associated with “hot” tumors enriched for cytotoxic/effector activity.^16^ In glycolysis, glucose can serve as a dominant energetic and biosynthetic substrate, while redox buffering is tightly coupled to glycolytic flux. A key node in this coupling is cystine/cysteine handling: cystine uptake and reduction to cysteine imposes substantial NADPH demand, reinforcing dependence on glucose-derived reducing power (e.g., pentose phosphate pathway activity) and supporting early glutathione-based stress buffering during cisplatin exposure.^3^^,^^17^

By contrast, the CR state is consistent with metabolic remodeling toward mitochondrial biogenesis and oxidative phosphorylation (OXPHOS), accompanied by increased reactive oxygen species (ROS) pressure and heightened reliance on antioxidant and anaplerotic pathways.^18^^,^^19^ Within this paradigm, glutamine-derived carbon can support TCA cycle flux and mitochondrial ATP production, and histidine catabolism can replenish glutamate pools and contribute to anaplerosis under oxidative stress.8 In parallel, increased availability and/or turnover of thiol-related substrates (e.g., cystine/cysteine) is compatible with elevated demand for glutathione and other thiol-based antioxidant systems required for redox homeostasis under drug-induced ROS.^3^^,^^17^ Taurine, while not a glutathione precursor, may still contribute to oxidative stress adaptation and cytoprotection in stressed tissues.

IMPACT’s feature stability across algorithms supports this biology-driven separation between states. Across models, BM_MDSC and circulating glutamine emerged as the most consistently prioritized discriminators, with recurrent contributions from SP_CD8^+^ Teff and the amino acids histidine, proline, taurine, and cystine (Figure 2B, Tables S4–S7). Directional trends provide additional interpretability (Figures 2C, 2D, and S1): CS animals show higher circulating glutamine/histidine/taurine together with higher lung CD8^+^ effector T cell signals, whereas CR animals show higher proline and cystine and a stronger myeloid skew (including expanded MDSCs), consistent with a systemic shift toward stress-adaptation programs that can be immunosuppressive.

Within this mechanistic framing, elevated cystine and proline in CR are consistent with broader amino acid/redox rewiring that supports mitochondrial function and ROS control.^3^^,^^17^ Concomitant MDSC expansion provides a plausible bridge between metabolic remodeling and immune suppression, as myeloid suppressor programs can impair CD8^+^ and NK effector activity through ROS-dependent mechanisms and nutrient depletion.^16^^,^^17^ Together, the integrated systemic signature captured by IMPACT links mitochondrial/ROS adaptation with a myeloid-skewed immune landscape that may reduce immune-mediated tumor control and thereby reinforce cisplatin resistance.

The prioritization of BM_MDSC and circulating glutamine by IMPACT suggests that myeloid suppressor expansion and amino acid availability are coupled at the organismal level. One mechanistically supported possibility is direct metabolic regulation, whereby glutamine availability and glutaminase-dependent pathways contribute to the generation and suppressive programming of immature myeloid cells/MDSC-like populations. In human cancer, glutamine availability and glutaminase activity have been reported to regulate the emergence of suppressive immature myeloid cells and their immunosuppressive phenotype.^10^ Consistent with this, pharmacologic targeting of glutamine metabolism has been shown to remodel suppressive myeloid compartments and enhance antitumor immunity.^13^ In our dataset, lower serum glutamine in CR animals may therefore reflect increased whole-body utilization by an expanded myeloid compartment and/or altered host nitrogen handling under drug-associated stress; importantly, serum concentrations report systemic availability rather than intratumoral flux.

A second, nonmutual exclusive explanation is coordinated regulation by upstream inflammatory cues that drive myelopoiesis and MDSC expansion in the bone marrow while concurrently reshaping systemic metabolism. Tumor and host-derived cytokines and growth factors (e.g., GM-CSF, IL-6, and related inflammatory programs) are well-established drivers of MDSC expansion and suppressive function.^20^ In patients with NSCLC, circulating MDSC-like populations have also been associated with poor response to chemotherapy,^21^ reinforcing the plausibility that a systemic myeloid axis is linked to treatment response states. Importantly, our current data are associative and cross-sectional; we therefore present this glutamine-MDSC coupling as a mechanistic *hypothesis* that motivates future perturbation studies (e.g., glutamine pathway inhibition or depletion models combined with functional MDSC assays) to establish causality.

### Signatures of cancer presence: *Cancer* vs*. No cancer*

We next asked whether systemic immunometabolic profiling can identify malignancy independent of cisplatin sensitivity, by pooling CS and CR animals into a single “Cancer” class and comparing this group with “*No Cancer*” controls (Figure 3, Tables S9–S17). Across algorithms, cancer status was classified with high accuracy, with the strongest discrimination achieved by nonlinear models (e.g., Random Forest), whereas logistic regression performed substantially worse. This divergence is consistent with the idea that cancer presence is encoded by interaction- and context-dependent structure across metabolites and organ-resolved immune populations, rather than by purely additive shifts in individual variables.

Importantly, interpretability again emerged from stability: feature rankings converged across models, and RFE preserved strong performance in reduced classifiers, supporting a compact, reproducible signature rather than high-dimensional idiosyncrasy. Within that compact axis, lung-granular MDSCs (LLG_MDSC) were repeatedly prioritized as the dominant cancer-associated discriminator, with phosphoserine also consistently ranked among the most informative metabolic features, and CD8^+^ Teff-related signals (e.g., SP_CD8^+^ Teff and lung/MLN CD8^+^ Teff features) contributing additional value in nonlinear models. Directional profiling reinforces this structure (Figures 3C–3F): relative to no cancer controls, CS animals show elevations in taurine, glutamine, serine, and histidine together with increased lung/MLN CD8^+^ Teff signals, whereas CR animals show increased ammonia/glutamate with pronounced phosphoserine elevation and stronger myeloid skewing (including BM_MDSC). Collectively, these coordinated patterns are consistent with the systemic remodeling of nitrogen handling and serine/one-carbon-linked metabolism coupled to early myeloid reprogramming as a hallmark of malignancy detectable at the organismal level.

Finally, these results underscore why multivariate, organ-aware modeling is especially valuable for systems immunometabolism. Univariate comparisons can identify marginal shifts (e.g., “phosphoserine differs” or “LLG_MDSC differs”), but they do not reconstruct the coupled, multiorgan structure by which malignancy is encoded, where the diagnostic meaning of any single metabolite can depend on concurrent immune context, and where multiple moderate effects can combine into a highly discriminative systemic state. By prioritizing features that recur across model classes and remain informative under feature reduction, IMPACT yields signatures that are both predictive and biologically interpretable, providing a structured map of the immune-metabolic circuitry associated with cancer presence (Figures 4A and 4B).

Together, these findings indicate that coordinated systemic immunometabolic remodeling contains reproducible information associated with both cisplatin sensitivity and cancer status. The IMPACT framework offers an interpretable approach to map these systemic patterns, identify compact feature signatures, and generate testable hypotheses linking circulating amino acid availability to organ-specific immune composition. The framework’s robustness is supported by strong cross-validated performance and by the stability of top discriminative features following covariate adjustment and recursive feature elimination (RFE), consistent with a compact and reproducible systemic signature rather than high-dimensional overfitting. By coupling predictive performance with biological interpretability, IMPACT highlights consistent, prioritizable signals across model classes that can be advanced into mechanistic validation and, ultimately, translation-focused assay design.

### Limitations of the study

Although the IMPACT framework achieved high accuracy in classifying cisplatin sensitivity and cancer status, several limitations outline clear priorities for future development and clinical translation.

(1) *Temporal resolution and tissue specificity of metabolomics:* The current metabolic readout is limited to circulating serum analytes collected at a single terminal time point. As a result, differences in serum abundance may reflect altered tissue uptake, production, or clearance rather than direct tumor-intrinsic metabolic flux. Future work should incorporate longitudinal sampling in spontaneous or genetically engineered lung cancer models to capture dynamic state transitions. In parallel, mechanistic follow-up studies, including intratumoral metabolite quantification and stable-isotope tracer experiments, will be necessary to determine whether the observed systemic differences represent causal drivers of response or compensatory host adaptations.
(2) *Immune functional characterization:* Our immune profiling primarily quantifies subset frequencies and therefore cannot resolve activation state, exhaustion, trafficking history, or cytotoxic competence. Accordingly, functional interpretations should be viewed as hypothesis-generating. Future iterations will expand the feature panel to include functional and differentiation markers and will incorporate complementary functional assays to directly assess exhaustion programs and suppressive mechanisms within the tumor microenvironment.
(3) Model generalizability and therapeutic scope: This study is based on a single experimental dataset using a syngeneic orthotopic lung tumor model and is restricted to cisplatin monotherapy. Independent external validation will be required to establish transportability and to quantify the impact of cohort effects and technical variability. More broadly, extending the framework across additional tumor genotypes and clinically relevant combination regimens (e.g., immune checkpoint blockade and radiation) will be important to distinguish drug-specific signatures from shared resistance programs. Although we mitigate overfitting risk through repeated resampling, covariate sensitivity analyses, and cross-algorithm feature stability (i.e., convergence of top-ranked predictors across distinct model classes), these internal checks do not replace validation in independent datasets. Future work will therefore focus on external validation and on sensitivity analyses that test the dependence of the reduced feature set on feature selection strategy.
(4) *Translational implementation and clinical applicability:* A key limitation is that the current feature set relies on terminal, multi-organ flow cytometry, which is not directly feasible in patients and also constrains longitudinal immune profiling in mice because peripheral blood volume limits repeated high-dimensional sampling. Nonetheless, the present work provides a conceptual blueprint for minimally invasive stratification. In this framework, bone marrow MDSCs are used as a discovery stage “mechanistic anchor” to localize a dominant site of myeloid expansion in the model, rather than to propose routine bone marrow sampling in clinical care. Clinically tractable implementations could instead be trained on peripheral blood surrogates (e.g., targeted flow panels capturing MDSC-like subsets and effector T cell states) together with serum metabolites. Human NSCLC studies support the clinical relevance of blood-based MDSCs, including associations with recurrence risk after surgery^11^ and meta-analytic evidence linking higher circulating MDSCs to worse outcomes across cohorts.^12^ In parallel, glutamine metabolism has been reported to regulate the generation and suppressive programming of immature myeloid cells,^10^ and therapeutic targeting of glutamine metabolism can remodel suppressive myeloid compartments and enhance anti-tumor immunity,^13^ providing a mechanistic rationale for evaluating serum glutamine together with peripheral myeloid phenotypes in future human IMPACT-style models. Prior to clinical deployment, the framework will require validation in independent human cohorts and rigorous control for host covariates, comorbidities, and treatment history.

## Resource availability

### Lead contact

Further information and requests for resources, reagents, data, or code should be directed to and will be fulfilled by the Lead Contact: Diane C. Lim (diane.lim@va.gov).

### Materials availability

This study did not generate new unique reagents. All cell lines, antibodies, and assay materials used in this work are commercially available or described within the STAR Methods and key resources table.

### Data and code availability

• Data: Flow cytometry and amino acid datasets are available in https://github.com/cbchenh/insilico.
• Code: IMPACT analysis code required to replicate the findings of this study is available in https://github.com/cbchenh/insilico.
• Other: All data supporting the findings are available in https://github.com/cbchenh/insilico.

## Acknowledgments

This work is supported by UM grant #PG013380 to C.B.C., by the 10.13039/100000050NHLBI #1R01HL142981-01A1 and Department of VA Merit review #1I01BX004872-01 to D.C.L., by Department of VA Merit review #1I01BX004371 to M.W.

## Author contributions

Conceptualization: E.Y.K.; methodology: E.Y.K.; software: C.B.C., Y.W., and A.P.; validation: C.B.C.; formal analysis: E.Y.K., D.C.L., C.B.C., and M.W.; investigation: E.Y.K., E.Q.K., and C.W.; resources: D.C.L., C.B.C., and M.W.; data curation: E.Y.K.; writing - original draft: E.Y.K. and D.C.L.; writing - review and editing: E.Y.K., D.C.L., C.B.C., and M.W.; visualization: E.Y.K. and D.C.L.; supervision: D.C.L., C.B.C., and M.W.; project administration: E.Y.K.; funding acquisition: D.C.L., C.B.C., and M.W.

## Declaration of interests

DCL serves on the medical advisory board of Apnimed, which has no direct conflict of interest with this study. DCL holds a patent related to the cell line used in this study. All other authors declare no competing interests.

## Declaration of generative AI and AI-assisted technologies in the writing process

During the preparation of this work, the authors utilized ChatGPT and Gemini to improve sentence clarity and linguistic flow. Following the use of these tools, the authors critically reviewed, verified, and edited the content as necessary. The authors maintain full responsibility for the accuracy and integrity of the final published article.

## STAR★Methods

### Key resources table

**Table:** 

REAGENT or RESOURCE	SOURCE	IDENTIFIER
**Antibodies**		

PE anti-mouse CD4, Clone GK1.5	BioLegend	Cat# 116005; RRID:AB_312697
EasySep™ Mouse CD49b Positive Selection Kit	STEMCELL Technologies	Cat# 18755; N/A
APC anti-mouse CD314 (NKG2D), Clone CX5	BioLegend	Cat# 130212; RRID:AB_1236372
Anti-mouse CD8a, Clone 536.7	BioLegend	Cat# 100708; RRID:AB_312747
EasySep™ Mouse TIL (CD45) Positive Selection Kit	STEMCELL Technologies	Cat# 1000350; N/A
Alexa Fluor 488 anti-mouse FOXP3, Clone 150D	BioLegend	Cat# 320012; RRID:AB_439748
APC/Fire 750 anti-mouse CD3, Clone 17A2	BioLegend	Cat# 100248; RRID:AB_2572118
EasySep™ Mouse PECD25 Positive Selection Kit	STEMCELL Technologies	Cat# 18782; N/A
APC/Fire 750 anti-mouse Ly6C, Clone HK1.4	BioLegend	Cat# 128046; RRID:AB_2616731
APC anti-mouse F4/80, Clone BM8	BioLegend	Cat# 123116; RRID:AB_893481
Anti-mouse Ly6G/Ly6C (Gr1), Clone RB68C5	BioLegend	Cat# 108408; RRID:AB_313373
FITC anti-mouse CD11b, Clone M1/70	BioLegend	Cat# 101206; RRID:AB_312789
PE antimouse IA/IE, Clone M5/114.15.2	BioLegend	Cat# 107608 RRID: AB_313323
Live/dead FVD violet	BioLegend	Cat# L34955; N/A

**Chemicals**		

Sulfosalicylic acid (3%)	Sigma	Cat# S2130
Amino acid internal standards	Biochrom	N/A
Biochrom 30+ Amino Acid Analysis Kit	Biochrom	N/A
Fc-block	BioLegend	Cat# 101320
TrueNuclear™ Transcription Factor Buffer Set	BioLegend	Cat# 424401

**Deposited data**		

Flow cytometry, raw FCS files	This paper	Zenodo (pending acceptance)
Amino acid data, normalized	This paper	Zenodo (pending acceptance)
IMPACT code	This paper	Zenodo (pending acceptance)

**Experimental models: Cell lines**		

SmKPP.1, Cisplatin Sensitive	Lim	U.S. patent No. 9,556,482
SmKPP.1, Cisplatin Resistant	Wangpaichitr	NA

**Experimental models: Organism/strain**		

C57BL/6J mice	The Jackson Laboratory	RRID: IMSR_JAX:000664
2% isoflurane in 1.5L/min oxygen	Patterson Veterinary	Cat# 078931389
Buprenorphine HCl Injection (0.3 mg/mL)	Par Pharmaceutical	Cat# 4202317905
Meloxicam (5 mg/mL Injection)	Norbrook Laboratories	Cat# 6000609271

**Software and algorithms**		

R v4.3.2	R Foundation	https://www.rproject.org
caret package v6.0	CRAN	https://cran.r-project.org/package=caret
XGBoost package v3.1.2.1	CRAN	https://cran.r-project.org/web/packages/xgboost/index.html
randomForest package v4.7-1.2	CRAN	https://cran.r-project.org/web/packages/randomForest/index.html
gbm package v2.2.2	CRAN	https://cran.r-project.org/web/packages/gbm/index.html
Prism v10	GraphPad	https://www.graphpad.com
CytExpert	Beckman Coulter	N/A
Biochrom 30+ Amino Acid Analyzer Software	Biochrom Ltd.	N/A
ITKsnap	Open source	http://www.itksnap.org

### Experimental model and study participant details

#### Cell lines

##### CS cell line

SmKPP.1 is a murine lung adenocarcinoma cell line we developed,^22^ derived from a male mouse and independently authenticated by the American Type Culture Collection (ATCC) using Short Tandem Repeat (STR) analysis (U.S. patent No. 9,556,482). The SmKPP.1 cells originated from a lung tumor induced by injecting Ad5SPCCre virus into the lung of a genetically modified mouse carrying Kras^G12D+^; Tp53 ^fl/fl^; myristoylated p110 ^fl/fl^ ROSA-gfp mutations.^23^ These clone cells reflect key genetic mutations commonly observed in human NSCLC, including KRAS (∼30% of all NSCLC cases),^24^ Tp53 inactivation,^25^ and PIK3CA mutations (6–10% of patients).^26^^,^^27^^,^^28^ As these mutations are significant pharmacological targets under investigation, SmKPP.1 serves as a clinically relevant model for studying lung adenocarcinoma.

#### CR cell line

Once authenticated, SmKPP.1 cells were made cisplatin resistant by treating the cells with clinical grade cisplatin provided by the Miami VA Hospital at concentrations maintaining half maximal inhibitory concentration (IC50). Parental, cisplatin sensitive, cells were seeded at 4x10^4^ in 6 well plates. Starting at 0.4 μg/mL cisplatin, the cells were treated and monitored daily until reaching a 2-fold increase in IC50 concentration which took approximately 12 weeks. The cells were left to recover from cisplatin toxicity then treated again with cisplatin at an increased concentration until the cells again reached a 2-fold increase in IC50 concentration. The process was repeated five times then maintained in media with 1 μg/mL of cisplatin. Cells were considered cisplatin resistant once they were confirmed to possess a 57-fold resistance to cisplatin. Establishing cisplatin resistant variants have been published previously.^19^

#### Mycoplasma testing

Mycoplasma testing was completed on both cell lines using the MycoAlert Mycoplasma Detection Kit (Lonza, Cat. no. LT07218) prior to the start of the experiment.

### Cell media and preparation

Cells are always preserved in liquid nitrogen vapor to prevent contamination. Upon thawing, cells were maintained in RPMI 1640 medium containing L-glutamine, HEPES, and Phenol Red (Thermofisher, Cat. no. 22400089) supplemented with 1% 10,000 U/mL penicillin, 10 mg/mL streptomycin (VWR, Cat. no. 3920406) and 10% fetal bovine serum (HyClone, Cat. no. SH30088.03) at 37°C in a 5% CO_2_ incubator and were passaged twice prior to experimental use.

#### Animal

All procedures were approved by the Miami VA IACUC (protocol #1581095) and conducted in accordance with institutional and federal guidelines. C57BL/6J mice (RRID:IMSR_JAX:000664; The Jackson Laboratory) were bred in-house. Breeding trios (2 females:1 male) were established at 8–12 weeks of age and maintained on a 12 h light/12 h dark cycle at 21°C–23°C and 40–60% humidity, with *ad libitum* access to food and water. Pregnant females were moved to larger hamster cages, housed in pairs, and were provided nestlets for added warmth. Pups were weaned and separated into same-sex cages in groups of five after four weeks.

#### Syngeneic orthotopic mouse model of lung adenocarcinoma

*Mice* were anesthetized with continuously inhaled 2% isoflurane in 1.5L/min oxygen via a nose cone. Analgesics (0.05 mg/kg buprenorphine and 5 mg/kg meloxicam) were administered subcutaneously prior to injections. SmKPP.1-CS or SmKPP.1-CR cells (15 μL volume, prepared as described in the cell culture section) or 1XPBS were injected directly into the left lung. Injection methods were based on previously described protocols.^22^^,^^29^

Mice were enrolled at 9–14 weeks (mean ∼13 weeks, correlating to 20–30 human years), balanced by sex, and randomized into four experimental cohorts.

• Cohort 1 (CS): orthotopic SmKPP.1CS cells; n = 73 (33 M, 40 F)
• Cohort 2 (CR): orthotopic SmKPP.1CR cells; n = 54 (27 M, 27 F)
• Cohort 3 (Control 1): 1XPBS intrathoracic injection; n = 29 (15 M, 14 F)
• Cohort 4 (Control 2): No injection; n = 30 (15 M, 15 F)
• Cohorts 3 + 4 were pooled as No Cancer after PERMANOVA validation (Table S9).

### Method details

#### Day of injection

On the day of injection, cells were washed using sterile 1XPBS (Thermofisher, Cat. no. 14190144), detached using 0.05% Trypsin (Corning, Cat. no. 25051Cl) and diluted with 3 times the volume of trypsin using RPMI 1640 (Thermofisher, Cat. no. 22400089) then centrifuged at 180 X g for 5 min at 25°C. Cells were checked for cell viability and total live cell count using a TC20 Automated Cell Counter (Bio-Rad, Cat. no. 1450102) in a 1:1 dilution of trypan blue (Corning, Cat. no. 25900Cl) then resuspended in 1XPBS such that a 15 μL aliquot provided 2.5x10^5^ cells. Aliquots were kept on ice until injection.

#### Tissue Collection

Following deep anesthesia with 2% isoflurane, pedal reflexes were tested to confirm the absence of pain response. Blood was drawn directly from the right ventricle using a 20G x 1/2″ 3 mL syringe and collected in 1.5 mL Eppendorf tubes for amino acid analysis. The left lung, mediastinal lymph nodes, spleen, and bone marrow were harvested and placed in 500 μL of 1X PBS for immediate flow cytometry.

#### Blood processing

Blood (∼700-1000uL per mouse) were allowed to clot at room temperature for at least 30 min but no longer than 1 h. The samples were centrifuged at 2,300 X g for 15 min at 4°C. Serum was collected and stored at −80°C until analysis.

#### Tissue processing

Tissues were minced and filtered through 40 μm strainers (Falcon #352340) in 1XPBS +2% FBS. BM was flushed from femurs/tibias (26 G needle). RBC lysis: 1% ammonium oxalate for 10 min at room temperature then quenched with 15 mL 1XPBS; spin at 1,000 X g, 5 min at 4°C; resuspend in 1 mL DPBS; counts via Bio-Rad TC20.

#### Flow cytometry

##### Sample preparation and staining

Tissue samples were minced, filtered through a 40 μm strainer (Falcon, Cat. no. 352340) with 1X PBS and centrifuged at 1000 X g for 5 min. Red blood cells were lysed with 1% ammonium oxalate, incubated for 10 min, and quenched with 15 mL 1X PBS. Cell pellets were resuspended in 1 mL 1X DPBS and split into four tubes for staining of.

• regulatory T cells (CD45+/CD4+/CD25+/FoxP3+)
• myeloid derived suppressor cells (F4/80^low^/Gr1+/Ly6C+/CD11b+)
• effector T cells (CD45+/CD3+/CD8+)
• natural killer cells (CD45+/CD49b+/CD3/NKG2D+)

All samples were incubated in 50uL of a 1:300 solution of Fc-block (BioLegend, Cat. no. 101320) and FACS buffer (1XPBS, 2% FBS, 1 mM EDTA) for 15 min. The cells were then stained with 1:100 of their corresponding conjugated antibodies at room temperature for 20 min in the dark. Cells set for regulatory T cell staining were further fixed and permeabilized with the TrueNuclear Transcription Factor Buffer Set according to the manufacturer’s protocol (BioLegend, cat no. 424401) for the staining of intracellular FOXP3. Flow cytometry was performed on a CytoFLEX (Beckman) flow cytometer and results were analyzed using CytExpert.

##### Cytometer and gating

Beckman Coulter (Figure S3) CytoFLEX (*Violet, Blue, and Red lasers*); single stain controls for compensation. Note: Fluorescence minus one (FMO) was used to identify gating boundaries. Gating strategy as follows.

• Mouse CD8^+^ T cells (Figure S3A). (i) Viable cells were gated based on CD45^+^ versus Fixable Viability Dye (FVD). (ii) CD3^+^CD8^+^ were gated from CD45+live. (iii) NKG2D+ and CD8^+^ cells were selected from CD3^+^CD8+population.
• Mouse NK cells (Figure S3A). (i) Viable cells were gated based on CD45^+^ versus Fixable Viability Dye (FVD). (ii) CD3^+^CD11b+ were gated from CD45+live. (iii) NKG2D+ and CD49b+ cells were selected from CD3^+^CD11b+population.
• Mouse MDSC cells (Figure S3B). (i) Viable cells were gated based on CD45^+^ versus Fixable Viability Dye (FVD). (ii) F4/80^low^ and Ly6C+ were gated from CD45+live. (iii) CD11b+ and Gr1+ were selected from the F4/80^low^ and Ly6C+ population.
• Mouse regulatory T cells (Figure S3B). (i) Viable cells were gated based on CD45^+^ versus Fixable Viability Dye (FVD). (ii) CD4^+^CD25^+^ were gated from CD45+live. (iii) CD4^+^ and intracellular FoxP3+ cells were selected from CD4^+^CD25^+^ population.

#### Amino acid profiling

##### Serum analysis

200 μL of 3% Sulfosalicylic Acid (Sigma, Cat. no. S2130) was added to 200 μL of serum, vortexed, and incubated in a 55°C heat block for 20 min. The samples were centrifuged again at 2500 X g for 20 min at 4°C. The supernatant was filtered through a 0.22 μm PES syringe filter (VWR, Cat. no. 76479024) and analyzed using the Biochrom 30+ Amino Acid Analyzer (Biochrom, Ltd., Cambridge, UK).

##### Data calibration

To ensure data consistency, a standard was run before each batch of samples and normalized to an internal standard of known concentration. Amino acid concentrations in each sample were calculated by comparing the peak area of an amino acid to the normalized standard’s peak area, followed by multiplication with its specific response factor derived from calibration.

### Quantification and statistical analysis

#### Machine learning environment

All statistical modeling and machine learning analyses were performed in R v4.3.2 (R Foundation for Statistical Computing) using the packages missForest, caret, dplyr, pROC, randomForest, xgboost, gbm, PRROC, and vip.

#### Preprocessing

Numerical variables were z-scaled so that each feature had mean 0 and variance 1 to reduce numerical instability. Missing values (1.43% of all entries across continuous and categorical variables) were imputed using the missForest algorithm to preserve nonlinear relationships. The analysis proceeded through three successive data objects, outlined in Supplement, Data S3/Methods S3, Table S18.

#### Model training and feature importance workflows

Gradient Boosting (GB), Random Forest (RF), XGBoost (XGB), and Logistic Regression (LR) models were trained on 80% of the data with 20% held out for testing, repeated across 30 random resampling iterations. Performance metrics included the area under the ROC curve (AUC), accuracy, sensitivity, specificity, precision, and F1 Score. Decision thresholds were optimized using a grid search over the range 0.05–1.00 in 0.01 increments, with F1score as the evaluation metric.

RFE was implemented as a backward selection procedure. For each algorithm, RFE began with the full initial predictor set and iteratively removed the lowest ranked feature based on model specific variable importance, averaged and rescaled to 0–100 across 30 independent 80/20 train-test splits. At every step, models were refit on the reduced feature set in each split, and performance was reestimated on the held out data using AUC as the optimization metric. Because AUC is threshold free, selection of the preferred feature subset for each algorithm was based on mean AUC across resamples and did not depend on any fixed classification cutoff. For the full initial models and, in the stepwise analyses, for each method subset combination, we pooled test set probabilities over all 30 splits and optimized the operating classification threshold by maximizing the F1 score over a grid of candidate cutoffs from 0.05 to 1.00 in 0.01 increments. Stability of feature rankings and elimination paths was characterized by aggregating importance profiles across resamples, and in separate full versus reduced feature comparisons, paired DeLong tests were used to assess differences in AUC.

Comparison of RFE vs. SHAP. As a feature-ranking sensitivity analysis, we computed SHAP feature-importance rankings for each model and compared them to the variable importance rankings used in the RFE workflow. Concordance for Step 1 between variable importance rankings and SHAP rankings was quantified using Spearman rank correlation and Top-N overlap (*N* = 10, 15, 20); concordance for Step 2 between RFE best AUC’s number of features and SHAP rankings was quantified using Spearman rank correlation and Top 10 overlap. Summary statistics are provided in the Supplement, Data S3/Methods S3, Table S19, for both CS vs. CR related to Figure 2 and Cancer vs. No Cancer related to Figure 3. The complete SHAP output spreadsheets are available via GitHub alongside the released analysis code.

#### Group level statistical comparisons

Global group differences in the 41dimensional immunometabolic profile were assessed using permutational multivariate analysis of variance (PERMANOVA) on the scaled features. To complement this global test, each feature was analyzed individually using one-way ANOVA with Group (1XPBS vs. No Inj) as the factor, and we report group means, F-statistics, raw *p*-values, Bonferroni adjusted *p*-values, Benjamini-Hochberg FDR-adjusted *p*-values, and partial η^2^ to quantify effect size (Table S9). As no significant multivariate separation was observed (PERMANOVA *p* > 0.05) and only small feature level effects were detected, the “1XPBS” and “No Injection” groups were combined into a single Control category for all subsequent Cancer vs. Control modeling tasks.

#### Organization of machine learning outputs

All machine learning outputs are stored as tabular files deposited with the dataset. For both classification problems (*CS* vs. *CR* and *Cancer* vs. *No Cancer*), the archive includes.

• Resampling level test set predictions and corresponding F1optimized operating thresholds;
• Per iteration performance metrics for each algorithm and feature set configuration;
• Model based variable importance profiles and backward elimination histories, including recursive feature elimination trajectories that summarize how discrimination changes as features are removed.
• For the “1XPBS” vs. “No injection” comparison, the archive additionally contains multivariate PERMANOVA results and per feature univariate statistics.

These outputs underlie the main performance figures and tables and support reproducibility and secondary analyses.

### Additional resources

No additional resources were generated.
