# Supplementary material for: Systemic immunometabolic profiling classifies cisplatin sensitivity states using interpretable machine learning
Source: iScience. 2026 Feb 17;29(3):115037. doi: 10.1016/j.isci.2026.115037 (PMC12993415; doi:10.1016/j.isci.2026.115037)
Supplement: Document S1. Figures S1–S3 and Tables S1–S19, and Data S1–S3 [file mmc1.pdf]

iScience, Volume *29*

Supplemental information

Systemic immunometabolic profiling classifies cisplatin sensitivity states using interpretable machine learning

Emily Y. Kim, Diane C. Lim, Yujie Wang, Edison Q. Kim, Chunjing Wu, Ankita Paul, Cheng-Bang Chen, and Medhi Wangpaichitr

Summary of Supplementary Data/Methods

1. **Data S1/Methods S1: IMPACT for cisplatin-sensitive vs cisplatin-resistant tumors**

IMPACT distinguished CS from CR tumors with high cross-validated performance, with Random Forest achieving the strongest discrimination in both the initial model (AUC 0.925 ± 0.058; Table S1) and the covariate adjusted full model (AUC 0.953 ± 0.040; Table S2). Importantly, performance remained strong after feature reduction (Random Forest AUC 0.950 ± 0.039; Table S8), indicating that cisplatin sensitivity may be encoded in a stable systemic signature rather than a high-dimensional artifact. Feature stability across algorithms converged on compact classifiers comprising BM_MDSC, SP_CD8$^+$ Teff, glutamine, histidine, and proline (Figure 2B; Tables S4-S7). Directional analyses (Figure 2C-D; Figure S1A) support these findings: CS mice showed higher glutamine, histidine, and LLG_CD8$^+$ Teff, consistent with an immune-killing, glycolysis-dominant state, whereas CR mice displayed higher proline, BM_MDSC, and SP_MDSC, consistent with an immune-suppressive, OXPHOS-dominant, ROS-buffering state. Figure S1B compares these marginal mean-difference patterns with the compact multivariate feature set prioritized by IMPACT.

2. **Data S2/Methods S2: IMPACT for cancer vs no-cancer**

IMPACT can be generalized to cancer detection. The Cancer vs No Cancer classifier was trained by pooling cisplatin-sensitive (CS) and cisplatin-resistant (CR) tumors into a single “Cancer” class and comparing them with the combined “No Cancer” control group. Models classified Cancer (CS+CR) vs No Cancer with high performance, with Random Forest again achieving the strongest discrimination in both the initial model (AUC 0.942 ± 0.047; Table S10) and covariate adjusted full model (AUC 0.967 ± 0.040; Table S11). Performance remained strong after feature reduction (Random Forest AUC 0.955 ± 0.032; Table S17), reinforcing that the presence of cancer may be encoded in a stable systemic signature. Feature stability across algorithms converged on compact classifiers comprising LLG_MDSC, LLG_CD8$^+$ Teff, and phosphoserine (Figure 3B; Tables S13-S16). For interpretability, directional mean-difference plots are shown separately for No Cancer vs CS and No Cancer vs CR (Figure 3C–F; Figure S2A). In these comparisons, CS relative to No Cancer showed elevations in LLG_MDSC and LLG_CD8$^+$ Teff together with increased taurine, glutamine, glutamate, serine, and histidine (Figure 3C and 3E), whereas CR relative to No Cancer showed elevations in LLG_MDSC and BM_MDSC together with increased ammonia and glutamate (Figure 3D and 3F). Figure S2B compares these marginal mean-difference patterns with the compact multivariate feature set prioritized by IMPACT.

3. **Data S3/Methods S3: QUANTIFICATION AND STATISTICAL ANALYSIS**

1. Data S1/Methods S1: IMPACT for cisplatin-sensitive vs cisplatin-resistant tumors

All cisplatin-sensitive (CS) and cisplatin-resistant (CR) cohorts were used to classify cisplatin sensitivity state and identify the most influential systemic biomarkers. For ease of navigation, Step 1 (Tables S1–S3) summarizes full-model performance and covariate sensitivity analyses, whereas Step 2 (Tables S4–S8) reports RFE trajectories and reduced-model performance.

Step 1: Train Model for CS vs CR

The initial 41-feature model, without covariates, was trained using four supervised machine learning algorithms, Gradient Boost, XGBoost, Logistic Regression, and Random Forest, and evaluated by AUC, sensitivity, specificity, Accuracy, F1 score and precision. All four algorithms performed well in the initial 41-feature model (Table S1). Random Forest achieved the highest AUC (0.925 ± 0.058; sensitivity 0.865 ± 0.108; specificity 0.844 ± 0.097; Accuracy 0.854 ± 0.081), while Gradient Boost, XGBoost, and Logistic Regression returned mean AUCs of 0.889 ± 0.069, 0.876 ± 0.063, and 0.705 ± 0.085, respectively (Table S1). Performance for all algorithms exceeded minimal discriminatory thresholds.

To evaluate clinically relevant factors, all algorithms were retrained with inclusion of age, gender, and time lapsed from tumor injection to tissue collection. The covariate-inclusive full model results are shown in Table S2. Performance patterns remained consistent, with RF again achieving the highest AUC (0.953 ± 0.040; sensitivity 0.898 ± 0.089; specificity 0.863 ± 0.099; Accuracy 0.880 ± 0.070) and Gradient Boost and XGBoost returning similarly strong AUCs of 0.906 ± 0.049 and 0.921 ± 0.047, respectively (Table S2). DeLong comparisons between initial and full models (Table S3) did not yield robust evidence of improvement, indicating that covariate inclusion did not materially change model discrimination.

Table S1. IMPACT: CS vs. CR: Step 1, Initial Model Performance, related to Figure 2

Algorithm	AUC	Accuracy	Sensitivity	Specificity	Precision	F1 Score
**Gradient Boost**	0.889 (±0.069)	0.825 (±0.072)	0.898 (±0.081)	0.752 (±0.103)	0.788 (±0.074)	0.837 (±0.066)
**XGBoost**	0.876 (±0.063)	0.785 (±0.071)	0.890 (±0.086)	0.681 (±0.111)	0.741 (±0.071)	0.806 (±0.064)
**Logistic Regression**	0.705 (±0.085)	0.686 (±0.089)	0.671 (±0.141)	0.702 (±0.105)	0.694 (±0.090)	0.677 (±0.104)
**Random Forest**	0.925 (±0.058)	0.854 (±0.081)	0.865 (±0.108)	0.844 (±0.097)	0.851 (±0.083)	0.854 (±0.081)

"±" represents Standard Deviation of the performance metrics calculated across 30 random resampling iterations. Threshold represents the classification probability cutoff optimized via grid search to maximize the F1 score: CR is the positive class.

Table S2. IMPACT: CS vs. CR: Step 1, Full Model Performance (with covariates), related to Figure 2

Algorithm	AUC	Accuracy	Sensitivity	Specificity	Precision	F1 Score
**Gradient Boost**	0.906 (±0.049)	0.844 (±0.065)	0.877 (±0.092)	0.810 (±0.096)	0.828 (±0.074)	0.848 (±0.064)
**XGBoost**	0.921 (±0.047)	0.843 (±0.061)	0.906 (±0.078)	0.779 (±0.106)	0.810 (±0.073)	0.852 (±0.055)
**Logistic Regression**	0.723 (±0.086)	0.697 (±0.081)	0.694 (±0.137)	0.700 (±0.095)	0.699 (±0.082)	0.691 (±0.099)
**Random Forest**	0.953 (±0.040)	0.880 (±0.070)	0.898 (±0.089)	0.863 (±0.099)	0.872 (±0.079)	0.882 (±0.069)

"±" represents Standard Deviation of the performance metrics calculated across 30 random resampling iterations. Threshold represents the classification probability cutoff optimized via grid search to maximize the F1 score: CR is the positive class.

Table S3. IMPACT: CS vs. CR: Step 1, Initial vs Full Model Comparison, related to Figure 2		
**Algorithm**	**Median_DeLong_p-value**	**Significance_Consensus**
**Gradient Boost**	0.528	Not Robust
**XGBoost**	0.230	Not Robust
**Logistic Regression**	0.365	Not Robust
**Random Forest**	0.282	Not Robust

Not Robust = evidence for a difference in model performance was not consistent across resampling iterations. This indicates that adding covariates (age, sex, and time from tumor injection to tissue collection) did not materially change discrimination between CS and CR; therefore, covariates were excluded from the subsequent RFE step.

Step 2: Recursive Feature Elimination for CS vs CR

Recursive feature elimination (RFE) was applied to iteratively remove the lowest-ranked feature, followed by retraining after each step. At each iteration, models were retrained and operating decision thresholds were re-optimized via grid search over 0.05–1.00 in 0.01 increments (F1 maximization) to compute threshold-dependent metrics. For example, in Table S4 (GB), the initial 41-feature list had a mean AUC of 0.877. When the feature with the lowest mean importance score, threonine (Thr), was removed and the list decreased to 40 features, the AUC became 0.895. RFE iteratively generated AUCs across feature counts (Table S4B). The optimal iteration for Gradient Boost occurred at iteration 30, with 12 features, producing a mean AUC = 0.923 (Table S4B). RFE was also performed for XGBoost (Table S5), Logistic Regression (Table S6), and Random Forest (Table S7). Across all algorithms, BM-MDSC, histidine, SP CD8$^+$ Teff, and glutamine consistently emerged as high-ranking features.

Table S4. IMPACT: CS vs. CR, Step 2, Recursive Feature Elimination, related to Figure 2

Gradient Boost (GB)

A. GB, initial model, feature list ranked by importance

Rank	Feature	Mean Importance	SD Importance
1	BM_MDSC	100	0
2	Gln	47.357	17.270
3	His	30.018	15.914
4	Pro	26.542	8.320
5	SP_CD8+ T eff	25.648	14.349
6	MLN_NK	19.094	11.753
7	LLG_CD8+ T eff	17.280	8.655
8	LLG_NK	15.869	9.362
9	LLG_MDSC	14.400	10.045
10	Phe	12.594	8.549
11	Cys	11.077	6.820
12	LLG_Treg	10.830	7.429
13	SP_MDSC	9.932	5.201
14	MLN_Treg	9.931	6.932
15	BM_Treg	9.744	5.414
16	Amm	8.369	4.503
17	Orn	7.943	6.206
18	MLN_CD8+ T eff	7.422	5.529
19	SP_NK	7.019	4.398
20	Glu	6.665	5.182
21	Ile	6.470	4.878
22	MLN_MDSC	6.292	5.596
23	BM_CD8+ T eff	6.165	4.422
24	Arg	6.097	4.204
25	SP_Treg	6.087	3.489
26	Urea	6.006	5.319
27	Taur	5.276	3.980
28	BM_NK	4.893	5.658
29	Leu	4.441	4.975
30	Citr	4.348	3.395
31	Ser	4.035	4.007
32	Lys	3.885	3.598
33	Phser	3.818	3.376
34	Tyr	3.787	3.130
35	Pea	3.408	3.379
36	Val	3.091	3.737
37	Ala	2.778	2.373
38	Asn	2.609	2.112
39	Gly	2.165	2.112
40	Met	1.352	1.343
**41**	**Thr**	**1.308**	**2.078**
Mean AUC=0.877 for 41 features			

B. GB, initial model, RFE

Iteration	# of features	Mean AUC	Remove lowest ranked feature
**1**	**41**	**0.877**	**Thr**
2	40	0.895	Gly
3	39	0.893	Met
4	38	0.901	Citr
5	37	0.896	Taur
6	36	0.878	Pea
7	35	0.895	Ala
8	34	0.896	Val
9	33	0.891	Lys
10	32	0.893	Asn
11	31	0.915	Phser
12	30	0.909	Urea
13	29	0.890	Tyr
14	28	0.904	BM_NK
15	27	0.900	Leu
16	26	0.899	Glu
17	25	0.912	Arg
18	24	0.911	Ser
19	23	0.921	MLN_MDSC
20	22	0.884	BM_CD8+ T eff
21	21	0.893	LLG_Treg
22	20	0.895	SP_Treg
23	19	0.898	MLN_CD8+ T eff
24	18	0.900	Ile
25	17	0.910	Orn
26	16	0.909	SP_NK
27	15	0.910	Cys
28	14	0.916	SP_MDSC
29	13	0.914	Amm
**30**	**12**	**0.923**	**MLN_Treg**
31	11	0.912	BM_Treg
32	10	0.907	Phe
33	9	0.907	LLG_MDSC
34	8	0.910	LLG_NK
35	7	0.911	LLG_CD8+ T eff
36	6	0.908	Pro
37	5	0.882	His
38	4	0.897	SP_CD8+ T eff
39	3	0.869	MLN_NK
40	2	0.829	Gln
41	1	0.733	BM_MDSC

C. GB, reduced model, feature list

Rank	Feature
**1**	**BM_MDSC**
**2**	**Gln**
**3**	**MLN_NK**
**4**	**SP_CD8+ T eff**
**5**	**His**
**6**	**Pro**
**7**	**LLG_CD8+ T eff**
**8**	**LLG_NK**
**9**	**LLG_MDSC**
**10**	**Phe**
**11**	**BM_Treg**
**12**	**MLN_Treg**
Mean AUC=0.923 for 12 features	

Table S5. IMPACT: CS vs. CR, Step 2, Recursive Feature Elimination, related to Figure 2

Extreme Gradient Boost (XGB)

A. XGB, initial model, feature list ranked by importance

Rank	Feature	Mean Importance	SD Importance
1	BM_MDSC	98.433	8.271
2	Gln	39.298	18.538
3	His	23.444	21.282
4	MLN_NK	18.143	13.368
5	SP_CD8+ T eff	17.981	10.776
6	Pro	17.279	9.413
7	MLN_Treg	17.072	11.581
8	SP_MDSC	12.865	7.872
9	LLG_CD8+ T eff	12.471	12.626
10	LLG_NK	10.057	6.507
11	LLG_Treg	9.741	9.289
12	Ile	9.600	9.154
13	Leu	8.537	7.841
14	Amm	8.487	7.142
15	LLG_MDSC	7.378	6.927
16	BM_CD8+ T eff	7.040	5.115
17	BM_Treg	6.682	6.098
18	Glu	5.441	4.775
19	Cys	5.189	5.278
20	BM_NK	4.240	5.205
21	Taur	4.207	4.186
22	Ala	3.695	4.007
23	Ser	3.692	3.757
24	Arg	3.610	4.166
25	Val	3.504	5.143
26	SP_NK	3.482	4.314
27	Phe	3.376	4.188
28	Orn	3.286	3.093
29	Asn	2.838	3.864
30	Urea	2.666	4.114
31	SP_Treg	2.621	2.875
32	Citr	2.483	2.147
33	Tyr	2.466	2.758
34	MLN_CD8+ T eff	1.900	2.523
35	MLN_MDSC	1.719	1.978
36	Lys	1.663	3.092
37	Phser	1.660	1.506
38	Pea	1.381	1.981
39	Met	1.281	1.767
40	Gly	1.000	1.690
**41**	**Thr**	**0.673**	**1.019**
Mean AUC=0.885 for 41 features			

B. XGB, initial model, RFE

Iteration	# of features	Mean AUC	Remove lowest ranked feature
**1**	**41**	**0.893**	**Met**
2	40	0.897	Gly
3	39	0.890	Pea
4	38	0.877	Thr
5	37	0.906	Val
6	36	0.897	Citr
7	35	0.881	Phser
8	34	0.893	Lys
9	33	0.902	Ser
10	32	0.901	Tyr
11	31	0.891	Taur
12	30	0.886	Urea
13	29	0.901	Asn
14	28	0.909	MLN_MDSC
15	27	0.910	SP_Treg
16	26	0.908	Orn
17	25	0.911	MLN_CD8+ T eff
18	24	0.926	Ala
19	23	0.925	SP_NK
**20**	**22**	**0.933**	**Arg**
21	21	0.917	Cys
22	20	0.930	Ile
23	19	0.921	Glu
24	18	0.908	BM_NK
25	17	0.908	BM_CD8+ T eff
26	16	0.933	Phe
27	15	0.928	LLG_NK
28	14	0.914	Amm
29	13	0.918	SP_MDSC
30	12	0.921	LLG_Treg
31	11	0.930	BM_Treg
32	10	0.908	LLG_MDSC
33	9	0.924	Leu
34	8	0.904	Pro
**35**	**7**	0.911	LLG_CD8+ T eff
36	6	0.922	MLN_Treg
37	5	0.883	His
38	4	0.889	MLN_NK
39	3	0.871	Gln
40	2	0.793	SP_CD8+ T eff
41	1	0.705	BM_MDSC

C. XGB, reduced model, feature list

Rank	Feature
**1**	**BM_MDSC**
**2**	**SP_CD8+ T eff**
**3**	**Gln**
**4**	**MLN_NK**
**5**	**His**
**6**	**MLN_Treg**
**7**	**LLG_CD8+ T eff**
**8**	**Pro**
**9**	**Leu**
**10**	**LLG_MDSC**
**11**	**BM_Treg**
**12**	**LLG_Treg**
**13**	**SP_MDSC**
**14**	**Amm**
**15**	**LLG_NK**
**16**	**Phe**
**17**	**BM_CD8+ T eff**
**18**	**BM_NK**
**19**	**Glu**
**20**	**Ile**
**21**	**Cys**
**22**	**Arg**
Mean AUC=0.933 for 22 features	

Table S6. IMPACT: CS vs. CR, Step 2, Recursive Feature Elimination, related to Figure 2

Logistic Regression (LR)

A. LR, initial model, feature list ranked by importance

Rank	Feature	Mean Importance	SD Importance
1	Glu	66.439	27.553
2	Lys	61.220	27.870
3	SP_MDSC	55.079	26.757
4	Amm	53.838	31.198
5	Gln	53.742	30.156
6	LLG_CD8+ T eff	53.126	32.000
7	Citr	52.906	27.129
8	SP_NK	51.462	34.952
9	BM_Treg	51.381	26.816
10	Ser	50.785	27.493
11	BM_NK	49.779	27.554
12	Orn	49.104	27.341
13	Urea	47.159	28.566
14	Gly	44.781	32.281
15	Met	44.112	27.705
16	MLN_CD8+ T eff	37.128	25.965
17	Pea	34.139	30.306
18	Ala	34.067	23.482
19	BM_CD8+ T eff	34.010	27.916
20	SP_Treg	33.998	23.469
21	Arg	33.087	24.078
22	Ile	32.725	28.157
23	LLG_Treg	32.334	27.149
24	Thr	31.844	26.978
25	Asn	31.275	23.886
26	Pro	31.144	26.887
27	Cys	30.449	27.355
28	Phe	27.971	25.577
29	LLG_MDSC	26.524	21.427
30	SP_CD8+ T eff	26.229	22.449
31	Leu	26.043	27.138
32	His	26.043	20.344
33	BM_MDSC	25.883	23.304
34	LLG_NK	25.576	26.688
35	Val	24.193	25.413
36	Tyr	23.653	22.107
37	Taur	23.242	18.730
38	MLN_Treg	22.300	20.203
39	Phser	21.589	22.638
40	MLN_MDSC	19.891	21.884
**41**	**MLN_NK**	**19.461**	**19.414**

Mean AUC=0.726 for 41 features

B. LR, initial model, RFE

Iteration	# of features	Mean AUC	Remove lowest ranked feature
**1**	**41**	**0.726**	**MLN_NK**
2	40	0.743	Phe
3	39	0.743	Phser
4	38	0.755	LLG_NK
5	37	0.780	His
6	36	0.765	MLN_Treg
7	35	0.772	Leu
8	34	0.771	Tyr
9	33	0.789	BM_MDSC
10	32	0.787	Pro
11	31	0.793	Val
12	30	0.817	Cys
13	29	0.819	MLN_MDSC
14	28	0.864	SP_CD8+ T eff
15	27	0.843	Pea
16	26	0.858	Thr
17	25	0.871	Met
18	24	0.875	LLG_MDSC
19	23	0.882	SP_Treg
20	22	0.857	MLN_CD8+ T eff
**21**	**21**	**0.897**	**Asn**
22	20	0.883	BM_Treg
23	19	0.884	Citr
24	18	0.881	Gly
25	17	0.890	Amm
26	16	0.879	Arg
27	15	0.875	Lys
28	14	0.882	Gln
29	13	0.880	Urea
30	12	0.841	Ile
31	11	0.870	LLG_Treg
32	10	0.873	BM_CD8+ T eff
33	9	0.837	BM_NK
34	8	0.811	SP_NK
35	7	0.810	LLG_CD8+ T eff
36	6	0.812	Orn
37	5	0.752	SP_MDSC
38	4	0.680	Glu
39	3	0.679	Taur
40	2	0.595	Ala
41	1	0.652	Ser

C. LR, reduced model, feature list

Rank	Feature
**1**	**Ser**
**2**	**Ala**
**3**	**Taur**
**4**	**Glu**
**5**	**SP_MDSC**
**6**	**Orn**
**7**	**LLG_CD8+ T eff**
**8**	**SP_NK**
**9**	**BM_NK**
**10**	**BM_CD8+ T eff**
**11**	**LLG_Treg**
**12**	**Ile**
**13**	**Urea**
**14**	**Gln**
**15**	**Lys**
**16**	**Arg**
**17**	**Amm**
**18**	**Gly**
**19**	**Citr**
**20**	**BM_Treg**
**21**	**Asn**

Mean AUC=0.897 for 21 features

Table S7. IMPACT: CS vs. CR, Step 2, Recursive Feature Elimination, related to Figure 2

Random Forest (RF)

A. RF, initial model, feature list ranked by importance

Rank	Feature	Mean Importance	SD Importance
1	BM_MDSC	100	0
2	Gln	54.783	9.758
3	SP_CD8+ T eff	49.240	11.174
4	His	48.005	10.975
5	Phe	31.931	7.131
6	Pro	27.128	8.427
7	SP_MDSC	25.765	7.059
8	LLG_MDSC	25.313	6.482
9	Ser	25.172	6.167
10	LLG_CD8+ T eff	25.133	6.581
11	MLN_NK	24.847	7.187
12	LLG_Treg	24.003	5.409
13	BM_NK	23.357	6.745
14	MLN_Treg	22.764	4.505
15	LLG_NK	20.801	6.214
16	MLN_MDSC	20.451	5.012
17	Leu	20.206	3.538
18	Cys	19.976	5.535
19	BM_CD8+ T eff	18.948	5.224
20	SP_NK	18.693	4.825
21	Amm	18.584	4.723
22	SP_Treg	17.544	3.109
23	BM_Treg	16.636	3.591
24	Glu	15.773	3.223
25	Val	15.606	3.803
26	Ile	15.459	3.942
27	Gly	14.954	3.181
28	Orn	13.998	3.582
29	Citr	13.959	2.101
30	Taur	13.596	4.220
31	Tyr	13.469	3.250
32	Asn	13.366	3.132
33	Ala	12.843	2.464
34	Lys	12.812	2.755
35	Arg	12.808	2.820
36	Pea	12.725	3.836
37	MLN_CD8+ T eff	12.522	2.616
38	Urea	12.478	2.242
39	Met	11.406	1.821
40	Thr	11.146	1.891
**41**	**Phser**	**10.965**	**2.101**
**Mean AUC=0.928 for 41 features**			

B. RF, initial model, RFE

Iteration	# of features	Mean AUC	Remove lowest ranked feature
**1**	**41**	**0.928**	**Phser**
2	40	0.917	Thr
3	39	0.919	Met
4	38	0.898	Pea
5	37	0.928	Arg
6	36	0.912	MLN_CD8+ T eff
7	35	0.906	Taur
8	34	0.915	Lys
9	33	0.934	Asn
10	32	0.926	Tyr
11	31	0.939	Urea
12	30	0.924	Ala
13	29	0.910	Citr
14	28	0.920	Orn
15	27	0.919	Ile
16	26	0.924	Gly
17	25	0.929	Val
18	24	0.927	BM_Treg
19	23	0.946	Glu
20	22	0.932	SP_Treg
**21**	**21**	**0.950**	**BM_CD8+ T eff**
22	20	0.928	SP_NK
23	19	0.931	Cys
24	18	0.934	MLN_MDSC
25	17	0.929	Amm
26	16	0.938	Leu
27	15	0.921	LLG_NK
28	14	0.927	LLG_MDSC
29	13	0.889	BM_NK
30	12	0.906	LLG_CD8+ T eff
31	11	0.911	Ser
32	10	0.910	LLG_Treg
33	9	0.923	SP_MDSC
34	8	0.922	MLN_Treg
35	7	0.920	Pro
36	6	0.915	Phe
37	5	0.886	MLN_NK
38	4	0.877	His
39	3	0.892	SP_CD8+ T eff
40	2	0.841	Gln
41	1	0.689	BM_MDSC

C. RF, reduced model, feature list

Rank	Feature
**1**	**BM_MDSC**
**2**	**Gln**
**3**	**SP_CD8+ T eff**
**4**	**His**
**5**	**MLN_NK**
**6**	**Phe**
**7**	**Pro**
**8**	**MLN_Treg**
**9**	**SP_MDSC**
**10**	**LLG_Treg**
**11**	**Ser**
**12**	**LLG_CD8+ T eff**
**13**	**BM_NK**
**14**	**LLG_MDSC**
**15**	**LLG_NK**
**16**	**Leu**
**17**	**Amm**
**18**	**MLN_MDSC**
**19**	**Cys**
**20**	**SP_NK**
**21**	**BM_CD8+ T eff**
**Mean AUC=0.950 for 21 features**	

Performance for the reduced model across all algorithms remained strong (Table S8). RF achieved the highest AUC (0.950 ± 0.039; sensitivity 0.892 ± 0.073; specificity 0.885 ± 0.068; Accuracy 0.889 ± 0.051), while Gradient Boost and XGBoost returned AUCs of 0.923 ± 0.040 and 0.933 ± 0.048, respectively. Logistic Regression remained lower but still above baseline (AUC 0.897 ± 0.056). Compared to RF's initial (AUC 0.925 ± 0.058; Table S1) and full models (AUC 0.953 ± 0.040; Table S2), the reduced RF model showed only modest changes in performance, confirming that a small subset of systemic features retains most predictive power while improving interpretability.

Table S8. IMPACT: CS vs. CR: Step 2, Reduced Model Performance (top AUC features), related to Figure 2								
Algorithm	# Features	AUC	Accuracy	Sensitivity	Specificity	Precision	F1 Score	Threshold
Gradient Boost	12	0.923 (±0.040)	0.835 (±0.049)	0.935 (±0.062)	0.735 (±0.094)	0.785 (±0.061)	0.851 (±0.042)	0.26
XGBoost	22	0.933 (±0.048)	0.868 (±0.062)	0.895 (±0.081)	0.840 (±0.089)	0.853 (±0.073)	0.871 (±0.061)	0.41
Logistic Regression	21	0.897 (±0.056)	0.822 (±0.069)	0.821 (±0.099)	0.823 (±0.093)	0.828 (±0.083)	0.820 (±0.070)	0.44
Random Forest	21	0.950 (±0.039)	0.889 (±0.051)	0.892 (±0.073)	0.885 (±0.068)	0.889 (±0.063)	0.888 (±0.052)	0.50

"±" represents Standard Deviation of the performance metrics calculated across 30 random resampling iterations. Threshold represents the classification probability cutoff optimized via grid search to maximize the F1 score: CR is the positive class.

To directly compare the narratives produced by traditional univariate statistics versus IMPACT based multivariate feature prioritization, we visualized systemic immunometabolic differences between CR and CS groups using both approaches. Figure S1A summarizes the direction of the CR vs CS mean difference across the 23 significant systemic immunometabolic features identified in Figure 2C-D. In parallel, Figure S1B summarizes the 21 features retained in the best performing IMPACT Random Forest classifier (highest AUC; Table S7). For Figure S1B, we intentionally display arrow directionality using the CR vs CS direction from Figure 2C-D, so that the comparison focuses on a key distinction: which features shift on average (univariate effect) versus which features are prioritized for discrimination in a multivariate model (predictive contribution conditional on other features). Several patterns emerge from this comparison.

First, the mean difference view places relatively greater visual emphasis on amino acid shifts, whereas IMPACT/RFE assigns comparatively greater weight to immune cell features, without contradicting the metabolite signal. In Figure S1A, univariate testing highlights features with the largest marginal (average) shifts between CR and CS, which often yields multiple significant amino acids that are partially correlated and may reflect shared underlying chemistry (e.g., redox- and nitrogen-related axes). In contrast, Figure S1B shows the features retained by RFE for the best-performing Random Forest model (Table S7), which is explicitly optimized for discrimination rather than for enumerating every shifted marker. Under that logic, a multivariate algorithm will often retain one or a few representative amino acids (capturing a shared correlated signal) while elevating immune populations that add orthogonal information about state. Importantly, this is not a conflict: IMPACT still recapitulates key metabolite signals that also appear in the mean difference analysis (e.g., glutamine and histidine), suggesting that the reduced set is not "ignoring metabolism," but rather compressing it into a minimally redundant subset that maximizes discrimination.

Second, despite different selection mechanics, both approaches converge on the same high level biological polarity, an "effector leaning" CS state versus a "suppressive/stress adapted" CR state, spanning immune and metabolic compartments. In the mean difference profiles, CS is characterized by higher $CD8^+$ effector T cell features (notably lung $CD8^+$ Teff signals) together with higher systemic availability of amino acids such as glutamine, histidine, and taurine, whereas CR shows higher cystine and proline coupled to expansion of MDSCs, especially in bone marrow (and also visible in other organs depending on the feature panel). Crucially, the fact that this same "effector vs suppressive" polarization is visible both as marginal shifts (Figure S1A) and as RFE-retained predictors (Figure S1B) argues that CS/CR separation reflects coordinated systemic remodeling rather than a single outlier feature. Put differently: univariate plots show the direction of many coordinated shifts, while IMPACT highlights which subset of those coordinated shifts is most consistently useful for classifying individual animals.

Third, the overlap between univariate signal and RFE retention highlights a compact "core discriminator" set that is stable across algorithms, while clarifying why other significant markers are dropped without being biologically "dismissed." Across models, BM_MDSC and serum glutamine repeatedly emerge as top ranked features, with additional stable contributors that commonly include SP_CD8⁺ Teff and histidine (and, depending on the algorithm, other metabolites like proline/taurine/cystine). This stability is reinforced by the observation that reduced models retain essentially the same discriminatory performance as full models (e.g., Random Forest reduced model AUC = 0.950), consistent with substantial redundancy in the full feature space. In this context, features that show detectable mean shifts but are not retained by RFE should be interpreted as correlated or conditionally redundant markers, their biology may still matter, but their information may already be captured by another retained feature (or only become informative in a specific subset of samples). This illustrates the value of pairing mean differences with IMPACT: Figure S1A and S1B motivate an integrated interpretation in which effect size (mean differences) and predictive contribution (multivariate prioritization) jointly guide which pathways and cell states are most plausible to pursue mechanistically.

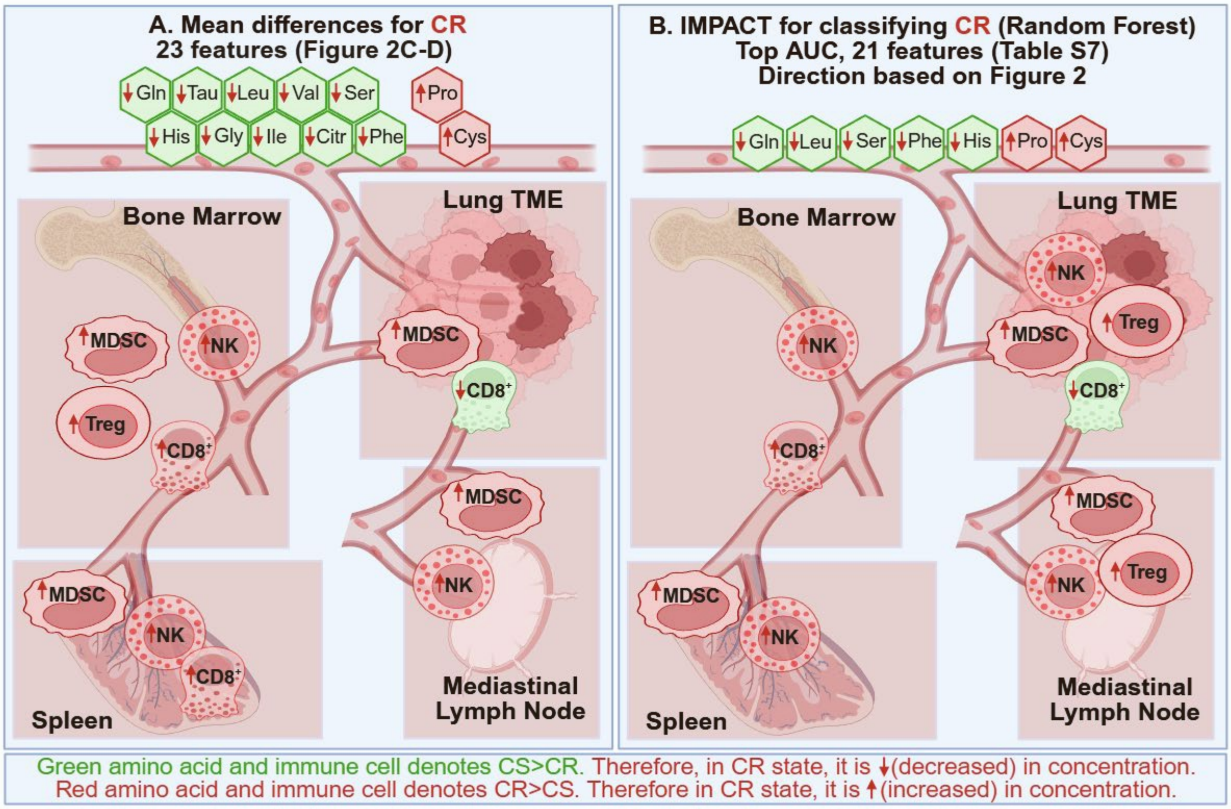

Figure S1. Systemic immunometabolic features comparing mean differences versus IMPACT to detect cisplatin resistance, related to Figure 2.

(A) Summary of the mean-difference profile (CR–CS) across the systemic immunometabolic features assayed in Figure 2C–D. Of the 41 total features, 23 met the significance criteria in Figure 2C–D and are shown here. Arrow direction indicates the direction of change in CR relative to CS (↑ higher in CR; ↓ lower in CR).

(B) Summary of the IMPACT feature prioritization for CR classification using recursive feature elimination (RFE) with a Random Forest model. The top-performing model (highest AUC; Table S7) retained 21 features, shown here. For direct comparison to univariate results, arrow direction indicates the CR vs CS directionality for each IMPACT-selected feature using the mean-difference direction derived from Figure 2C–D (↑ higher in CR; ↓ lower in CR).

2. Data S2/Methods S2: IMPACT for cancer vs no-cancer

All experimental data were used to classify cancer status and determine the most influential systemic biomarkers. This section first validates the pooled No Cancer controls (Table S9), then presents Step 1 full-model performance and covariate analyses (Tables S10–S12) and Step 2 RFE trajectories and reduced-model performance (Tables S13–S17). CS and CR cohorts were pooled into a broad "Cancer" group. To ensure experimental handling did not confound cancer detection, Cohort 3 (1XPBS) and Cohort 4 (No Injection) were compared. A permutational multivariate analysis of variance (PERMANOVA) including 41 metabolic and immune variables showed no significant overall effect of Outcome (1XPBS vs No Inj; pseudo-F = [F], R^2 = [R^2], p = [p]). Follow-up univariate ANOVAs with Bonferroni and FDR correction for the 41 tests did not identify any individual variable with a significant group difference (all corrected $p > 0.05$; Values shown represent group means derived from z-scaled features (mean = 0, variance = 1); 1XPBS and No Inj reported as mean. Table S9). Effect sizes were small (partial $\eta^2 \leq 0.11$), consistent with the absence of robust, marker-specific differences between the two groups. Collectively this supports their combination into a unified "No Cancer" control (Table S9).

Table S9. IMPACT: 1XPBS versus No Injection, related to Figure 3							
Feature	1XPBS	No Inj	F_value	P (raw)	P_Bonf	P_FDR(BH)	partial η²
Pro	-0.01	0.11	0.23	0.634	1	0.804	0.004
Phser	-0.49	0.12	4.57	0.0368	1	0.251	0.074
Taur	-0.14	-0.16	0.01	0.907	1	0.954	0
Pea	-0.17	-0.41	2.08	0.155	1	0.397	0.035
Urea	0.02	0.49	3.66	0.0606	1	0.259	0.06
Thr	-0.03	-0.02	0	0.978	1	0.999	0
Ser	-0.3	0.18	7.02	0.0104	0.427	0.222	0.11
Asn	-0.26	0.22	3.59	0.0632	1	0.259	0.059
Glu	-0.17	-0.55	5.15	0.0271	1	0.222	0.083
Gln	-0.1	-0.14	0.07	0.791	1	0.877	0.001
Gly	-0.24	0.07	0.91	0.344	1	0.583	0.016
Ala	-0.28	-0.02	1.58	0.214	1	0.487	0.027
Citr	0.17	0.48	1.25	0.269	1	0.576	0.021
Val	-0.09	0.24	2.63	0.11	1	0.323	0.044
Cys	-0.19	0.32	5.84	0.0189	0.773	0.222	0.093
Met	-0.08	0.13	0.63	0.43	1	0.66	0.011
Ile	0.03	0.12	0.17	0.686	1	0.804	0.003
Leu	-0.11	0.32	3.42	0.0695	1	0.259	0.057
Tyr	-0.07	0.22	1.09	0.301	1	0.576	0.019
Phe	-0.17	0.09	6.5	0.0135	0.553	0.222	0.102
Amm	-0.16	-0.36	2.65	0.109	1	0.323	0.044
Orn	-0.13	-0.37	0.95	0.334	1	0.583	0.016
Lys	-0.01	-0.01	0	0.999	1	0.999	0
His	-0.14	0.22	3.61	0.0624	1	0.259	0.06
Arg	0.12	0.26	0.32	0.572	1	0.804	0.006
LLG_Treg	0.35	-0.06	2.39	0.127	1	0.348	0.04
MLN_Treg	0.4	0.18	0.62	0.435	1	0.66	0.011
SP_Treg	0.08	-0.29	4.08	0.0481	1	0.259	0.067
BM_Treg	0.1	-0.07	0.25	0.622	1	0.804	0.004
LLG_CD8+ T eff	-0.23	-0.4	1.72	0.195	1	0.47	0.029
MLN_CD8+ T eff	-0.12	-0.44	2.76	0.102	1	0.323	0.046
SP_CD8+ T eff	0.4	0.27	0.46	0.498	1	0.73	0.008
BM_CD8+ T eff	0.35	0.44	0.1	0.757	1	0.862	0.002
LLG_NK	-0.04	-0.52	5.4	0.0237	0.971	0.222	0.087
MLN_NK	-0.07	-0.34	1.05	0.309	1	0.576	0.018
SP_NK	0.05	-0.2	0.87	0.356	1	0.583	0.015
BM_NK	0.3	0.15	0.22	0.64	1	0.804	0.004
LLG_MDSC	-0.47	-0.5	0.17	0.685	1	0.804	0.003
MLN_MDSC	-0.12	-0.09	0.03	0.872	1	0.941	0
SP_MDSC	0.08	-0.09	1.12	0.294	1	0.576	0.019
BM_MDSC	-0.01	0.06	0.19	0.663	1	0.804	0.003

Step 1: Train Model for Cancer vs No Cancer

Using the same 41-feature dataset, the initial model (no covariates) was trained across the four algorithms and evaluated by AUC, sensitivity, specificity, Accuracy, F1 score, and precision (Table S10). All algorithms performed above baseline discriminatory thresholds, with Random Forest achieving the highest AUC (0.942 ± 0.07; sensitivity 0.965 ± 0.034; specificity 0.715 ± 0.126; Accuracy 0.901 ± 0.037; F1 0.935 ± 0.023; precision 0.909 ± 0.036), while Gradient Boost, XGBoost, and Logistic Regression returned mean AUCs of 0.938 ± 0.045, 0.923 ± 0.055, and 0.783 ± 0.110, respectively (Table S10).

To evaluate clinically relevant factors, all algorithms were retrained with inclusion of age, gender, and time lapsed from tumor injection to tissue collection. The covariate-inclusive full model results are summarized in Table S11. Performance patterns remained consistent and improved relative to the initial model: Gradient Boost achieved an AUC of 0.966 ± 0.036 (Accuracy 0.915 ± 0.042), XGBoost achieved an AUC of 0.968 ± 0.033 (Accuracy 0.916 ± 0.045), Logistic Regression achieved an AUC of 0.805 ± 0.068 (Accuracy 0.805 ± 0.061), and Random Forest achieved an AUC of 0.967 ± 0.040 (Accuracy 0.929 ± 0.031) (Table S11). DeLong comparisons between initial and full models (Table S12) did not yield robust evidence of improvement, indicating that covariate inclusion did not materially change model discrimination.

Table S10. IMPACT: Cancer vs No Cancer: Step 1, Initial Model Performance, related to Figure 3

Algorithm	AUC	Accuracy	Sensitivity	Specificity	Precision	F1 Score
**Gradient Boost**	0.938 (±0.045)	0.891 (±0.058)	0.930 (±0.042)	0.779 (±0.150)	0.926 (±0.048)	0.927 (±0.038)
**XGBoost**	0.923 (±0.055)	0.860 (±0.049)	0.971 (±0.033)	0.536 (±0.154)	0.861 (±0.041)	0.912 (±0.030)
**Logistic Regression**	0.783 (±0.110)	0.771 (±0.066)	0.835 (±0.084)	0.585 (±0.145)	0.856 (±0.042)	0.843 (±0.051)
**Random Forest**	0.942 (±0.047)	0.901 (±0.037)	0.965 (±0.034)	0.715 (±0.126)	0.909 (±0.036)	0.935 (±0.023)

"±" represents Standard Deviation of the performance metrics calculated across 30 random resampling iterations. Threshold represents the classification probability cutoff optimized via grid search to maximize the F1 score: Cancer is the positive class.

Table S11. IMPACT: Cancer vs No Cancer: Step 1, Full Model Performance (With Covariates), related to Figure 3

Algorithm	AUC	Accuracy	Sensitivity	Specificity	Precision	F1 Score
**Gradient Boost**	0.966 (±0.036)	0.915 (±0.042)	0.985 (±0.023)	0.709 (±0.144)	0.910 (±0.041)	0.945 (±0.026)
**XGBoost**	0.968 (±0.033)	0.916 (±0.045)	0.972 (±0.033)	0.752 (±0.147)	0.921 (±0.044)	0.945 (±0.029)
**Logistic Regression**	0.805 (±0.068)	0.805 (±0.061)	0.832 (±0.070)	0.724 (±0.130)	0.899 (±0.044)	0.863 (±0.045)
**Random Forest**	0.967 (±0.040)	0.929 (±0.031)	0.988 (±0.021)	0.761 (±0.125)	0.925 (±0.037)	0.954 (±0.020)

"±" represents Standard Deviation of the performance metrics calculated across 30 random resampling iterations. Threshold represents the classification probability cutoff optimized via grid search to maximize the F1 score: Cancer is the positive class.

Table S12. IMPACT: Cancer vs No Cancer: Step 1, Initial vs Full Model Comparison, related to Figure 3

Algorithm	Median_DeLong_p-value	Significance_Consensus
**Gradient Boost**	0.352	Not Robust
**XGBoost**	0.222	Not Robust
**Logistic Regression**	0.379	Not Robust
**Random Forest**	0.301	Not Robust

Not Robust = evidence for a difference in model performance was not consistent across resampling iterations. This indicates that adding covariates (age, sex, and time from tumor injection to tissue collection) did not materially change discrimination between Cancer and No Cancer; therefore, covariates were excluded from the subsequent RFE step.

Step 2: Recursive Feature Elimination for Cancer vs No Cancer

RFE was applied as in the CS vs CR analysis, sequentially removing low-importance features; at each iteration, models were retrained and operating decision thresholds were re-optimized via grid search over 0.05–1.00 in 0.01 increments (F1 maximization). The initial 41-feature list ranked by importance (Table S13A) served as the starting point; at each step, the lowest-ranked feature (beginning with methionine) was removed, and models were retrained on the remaining features. The full sequence of feature counts and corresponding AUCs is summarized in Table S13B. The highest AUC occurred in iteration 32 using a 10-feature subset (Table S13C). RFE was also performed for XGBoost (Table S14), Logistic Regression (Table S15), and Random Forest (Table S16). Across all algorithms, phosphoserine, LLG_MDSC, and SP_CD8^{+} Teff consistently ranked among the most informative features. These mirrored the mean-difference directions observed in the main Figure 3C-F, supporting IMPACT's ability to distinguish cancer from No Cancer using systemic immunometabolic signals.

Table S13. IMPACT: No Cancer vs Cancer,
Step 2, Recursive Feature Elimination, related to Figure 3
Gradient Boost (GB)

A. GB, initial model, feature list ranked by importance

Rank	Feature	Mean Importance	SD Importance
1	LLG_MDSC	92.479	13.257
2	Phser	74.709	22.661
3	LLG_CD8+ T eff	58.786	21.232
4	MLN_NK	38.431	14.928
5	SP_CD8+ T eff	38.243	17.512
6	Citr	32.213	13.118
7	Urea	29.935	13.147
8	BM_NK	28.377	16.578
9	BM_CD8+ T eff	28.151	11.706
10	MLN_Treg	27.798	13.316
11	SP_MDSC	26.625	13.788
12	Orn	23.531	13.881
13	MLN_MDSC	19.949	10.414
14	LLG_NK	19.345	11.607
15	LLG_Treg	16.349	9.132
16	BM_MDSC	16.158	12.076
17	SP_NK	15.880	9.797
18	MLN_CD8+ T eff	15.357	10.847
19	Gln	15.191	10.157
20	Arg	14.974	11.147
21	Glu	13.851	10.345
22	His	11.770	10.207
23	Pea	9.462	11.502
24	BM_Treg	9.172	6.560
25	Cys	8.138	4.566
26	Lys	6.460	6.392
27	Amm	6.451	6.562
28	Asn	5.728	5.496
29	Tyr	4.956	4.068
30	Taur	4.874	4.610
31	Phe	4.823	7.323
32	Ser	4.258	5.171
33	Gly	4.104	3.321
34	SP_Treg	3.776	3.174
35	Pro	3.179	3.677
36	Val	2.777	3.525
37	Thr	2.545	3.266
38	Ala	2.331	3.173
39	Met	1.686	1.979
40	Ile	1.395	2.452
**41**	**Leu**	**1.148**	**1.966**
**Mean AUC=0.929 for 41 features**			

B. GB, initial model, RFE

Iteration	# of features	Mean AUC	Remove lowest ranked feature
**1**	**41**	**0.929**	**Leu**
2	40	0.921	Ala
3	39	0.927	Ile
4	38	0.940	Met
5	37	0.932	Val
6	36	0.946	Thr
7	35	0.939	Pro
8	34	0.934	SP_Treg
9	33	0.944	Tyr
10	32	0.931	Taur
11	31	0.937	Phe
12	30	0.939	Lys
13	29	0.934	Gly
14	28	0.946	Asn
15	27	0.936	Amm
16	26	0.930	Ser
17	25	0.941	Glu
18	24	0.952	BM_Treg
19	23	0.938	Pea
20	22	0.951	Gln
21	21	0.945	LLG_Treg
22	20	0.936	Arg
23	19	0.935	His
24	18	0.950	Cys
25	17	0.949	BM_MDSC
26	16	0.935	SP_NK
27	15	0.940	MLN_CD8+ T eff
28	14	0.952	MLN_MDSC
29	13	0.938	LLG_NK
30	12	0.940	BM_CD8+ T eff
31	11	0.938	Urea
**32**	**10**	**0.955**	**SP_MDSC**
33	9	0.931	Orn
34	8	0.922	BM_NK
35	7	0.914	Citr
36	6	0.916	MLN_Treg
37	5	0.907	MLN_NK
38	4	0.881	SP_CD8+ T eff
39	3	0.861	Phser
40	2	0.866	LLG_CD8+ T eff
41	1	0.709	LLG_MDSC

C. GB, reduced model, feature list

Rank	Feature
**1**	**LLG_MDSC**
**2**	**LLG_CD8+ T eff**
**3**	**Phser**
**4**	**SP_CD8+ T eff**
**5**	**MLN_NK**
**6**	**MLN_Treg**
**7**	**Citr**
**8**	**BM_NK**
**9**	**Orn**
**10**	**SP_MDSC**
**Mean AUC=0.955 for 10 features**	

Table S14. IMPACT: No Cancer vs Cancer, Step 2, Recursive Feature Elimination, related to Figure 3

Extreme Gradient Boost (XGB)

A. XGB, initial model, feature list ranked by importance

Rank	Feature	Mean Importance	SD Importance
1	LLG_MDSC	92.136	16.930
2	LLG_CD8+ T eff	69.389	25.260
3	Phser	57.163	23.014
4	SP_CD8+ T eff	35.773	15.481
5	MLN_NK	35.076	19.013
6	MLN_MDSC	29.593	21.086
7	Citr	28.464	13.022
8	BM_CD8+ T eff	24.520	11.931
9	BM_NK	18.744	9.887
10	SP_MDSC	16.652	13.453
11	Urea	15.993	14.312
12	Gln	14.856	11.837
13	MLN_Treg	14.077	9.212
14	Pea	13.847	10.971
15	BM_MDSC	13.816	12.276
16	LLG_NK	13.807	7.661
17	SP_NK	13.331	11.520
18	MLN_CD8+ T eff	12.574	10.557
19	Orn	11.426	11.131
20	Asn	10.627	10.427
21	Glu	10.517	10.659
22	Amm	10.452	11.928
23	His	9.692	8.310
24	Arg	9.240	7.843
25	BM_Treg	8.156	5.838
26	LLG_Treg	7.496	7.579
27	Ser	7.370	5.987
28	Gly	6.466	7.135
29	Cys	5.279	4.630
30	Taur	3.815	4.044
31	Pro	3.564	4.662
32	Tyr	2.411	3.486
33	Phe	2.302	3.502
34	Val	2.179	3.297
35	SP_Treg	2.029	3.841
36	Leu	1.715	3.210
37	Met	1.436	2.702
38	Lys	1.335	2.024
39	Ile	1.139	1.702
40	Thr	1.025	1.616
**41**	**Ala**	**0.932**	**2.305**

Mean AUC=0.922 for 41 features

B. XGB, initial model, RFE

Iteration	# of features	Mean AUC	Remove lowest ranked feature
**1**	**41**	**0.922**	**Met**
2	40	0.940	Ile
3	39	0.936	Thr
**4**	**38**	**0.952**	**Leu**
5	37	0.925	Ala
6	36	0.931	Pro
7	35	0.927	Val
8	34	0.943	Phe
9	33	0.951	SP_Treg
10	32	0.940	Lys
11	31	0.938	Tyr
12	30	0.944	Taur
13	29	0.942	Gly
14	28	0.937	Cys
15	27	0.937	Amm
16	26	0.947	BM_Treg
17	25	0.948	Asn
18	24	0.934	LLG_Treg
19	23	0.932	Ser
20	22	0.934	Pea
21	21	0.940	SP_NK
22	20	0.948	Arg
23	19	0.934	Glu
24	18	0.934	MLN_CD8+ T eff
25	17	0.946	Gln
26	16	0.941	SP_MDSC
27	15	0.939	LLG_NK
28	14	0.940	MLN_Treg
29	13	0.935	BM_CD8+ T eff
30	12	0.941	His
31	11	0.933	Urea
32	10	0.923	Orn
33	9	0.934	MLN_MDSC
34	8	0.920	BM_NK
35	7	0.934	MLN_NK
36	6	0.913	Citr
37	5	0.898	SP_CD8+ T eff
38	4	0.890	BM_MDSC
39	3	0.856	Phser
40	2	0.817	LLG_CD8+ T eff
41	1	0.722	LLG_MDSC

C. XGB, reduced model, feature list

Rank	Feature
**1**	**LLG_MDSC**
**2**	**LLG_CD8+ T eff**
**3**	**Phser**
**4**	**BM_MDSC**
**5**	**SP_CD8+ T eff**
**6**	**Citr**
**7**	**BM_NK**
**8**	**MLN_MDSC**
**9**	**Orn**
**10**	**Urea**
**11**	**His**
**12**	**BM_CD8+ T eff**
**13**	**MLN_Treg**
**14**	**LLG_NK**
**15**	**SP_MDSC**
**16**	**Gln**
**17**	**MLN_CD8+ T eff**
**18**	**Glu**
**19**	**Arg**
**20**	**SP_NK**
**21**	**Pea**
**22**	**Ser**
**23**	**LLG_Treg**
**24**	**Asn**
**25**	**BM_Treg**
**26**	**Cys**
**27**	**Gly**
**28**	**Taur**
**29**	**Tyr**
**30**	**Orn**
**31**	**Lys**
**32**	**SP_Treg**
**33**	**Phe**
**34**	**Tyr**
**35**	**Val**
**36**	**Ala**
**37**	**Leu**
**38**	**Thr**

Mean AUC=0.952 for 38 features

Table S15. IMPACT: No Cancer vs Cancer,
Step 2, Recursive Feature Elimination, related to Figure 3
Logistic Regression (LR)

A. LR, initial model, feature list ranked by importance				B. LR, initial model, RFE				C. LR, reduced model, feature list	
**Rank**	**Feature**	**Mean Importance**	**SD Importance**	**Iteration**	**# of features**	**Mean AUC**	**Remove lowest ranked feature**	**Rank**	**Feature**
1	LLG_MDSC	87.268	19.797	**1**	**41**	**0.784**	**Ala**	**1**	**LLG_MDSC**
2	Citr	82.463	23.277	2	40	0.769	Phe	**2**	**SP_CD8+ T eff**
3	SP_CD8+ T eff	82.138	17.793	3	39	0.799	Pro	**3**	**MLN_CD8+ T eff**
4	Phser	70.008	19.785	4	38	0.799	Orn	**4**	**Citr**
5	Urea	67.958	21.941	5	37	0.805	MLN_MDSC	**5**	**LLG_NK**
6	His	67.489	19.654	6	36	0.829	BM_NK	**6**	**LLG_CD8+ T eff**
7	LLG_CD8+ T eff	67.478	17.806	7	35	0.855	Ile	**7**	**Phser**
8	SP_MDSC	60.270	27.415	8	34	0.850	Glu	**8**	**Urea**
9	Tyr	60.059	30.298	9	33	0.883	MLN_NK	**9**	**Thr**
10	MLN_CD8+ T eff	57.596	29.054	10	32	0.861	Met	**10**	**Asn**
11	Leu	56.149	24.934	11	31	0.877	Lys	**11**	**LLG_Treg**
12	SP_NK	52.506	24.292	12	30	0.888	SP_Treg	**12**	**SP_MDSC**
13	Thr	48.812	23.246	13	29	0.880	Ser	**13**	**His**
14	BM_CD8+ T eff	48.329	27.022	14	28	0.896	Gly	**14**	**Leu**
15	LLG_Treg	47.175	28.360	15	27	0.888	BM_Treg	**15**	**SP_NK**
16	LLG_NK	46.764	24.917	16	26	0.909	BM_MDSC	**16**	**Tyr**
17	Asn	44.615	26.538	17	25	0.886	Pea	**17**	**BM_CD8+ T eff**
18	Taur	39.079	21.096	**18**	**24**	**0.917**	**MLN_Treg**	**18**	**Amm**
19	Gln	37.604	25.886	19	23	0.898	Cys	**19**	**Gln**
20	MLN_Treg	36.108	26.961	20	22	0.900	Val	**20**	**Arg**
21	Arg	35.729	23.301	21	21	0.915	Taur	**21**	**Taur**
22	Amm	35.453	25.321	22	20	0.895	Arg	**22**	**Val**
23	BM_Treg	34.526	27.633	23	19	0.910	Gln	**23**	**Cys**
24	BM_MDSC	33.265	24.660	24	18	0.906	Amm	**24**	**MLN_Treg**
25	Ser	32.852	24.080	25	17	0.905	BM_CD8+ T eff	**Mean AUC=0.917 for 24 features**	
26	Pea	32.671	27.779	26	16	0.891	Tyr		
27	SP_Treg	32.560	22.682	27	15	0.895	SP_NK		
28	Lys	31.390	24.117	28	14	0.905	Leu		
29	Met	31.126	24.936	29	13	0.895	His		
30	Val	30.931	26.568	30	12	0.901	SP_MDSC		
31	Gly	30.780	26.501	31	11	0.884	LLG_Treg		
32	Cys	29.410	22.923	32	10	0.874	Asn		
33	Glu	29.365	24.790	33	9	0.875	Thr		
34	MLN_NK	27.698	23.749	34	8	0.885	Urea		
35	MLN_MDSC	27.610	24.499	35	7	0.878	Phser		
36	Pro	25.239	22.093	36	6	0.873	LLG_CD8+ T eff		
37	BM_NK	24.882	25.964	37	5	0.866	LLG_NK		
38	Phe	24.679	24.150	38	4	0.826	Citr		
39	Ile	24.162	20.976	39	3	0.799	MLN_CD8+ T eff		
40	Orn	21.065	23.022	40	2	0.762	SP_CD8+ T eff		
**41**	**Ala**	**20.121**	**20.669**	41	1	0.713	LLG_MDSC		
**Mean AUC=0.784 for 41 features**									

Table S16. IMPACT: No Cancer vs Cancer, Step 2, Recursive Feature Elimination, related to Figure 3

Random Forest (RF)

A. RF, initial model, feature list ranked by importance

Rank	Feature	Mean Importance	SD Importance
1	LLG_MDSC	95.284	7.657
2	Phser	87.809	13.066
3	LLG_CD8+ T eff	53.422	13.574
4	BM_CD8+ T eff	48.795	10.282
5	SP_CD8+ T eff	48.456	13.856
6	MLN_NK	45.297	11.512
7	BM_NK	43.218	10.597
8	Orn	41.072	13.197
9	Citr	40.375	9.725
10	BM_MDSC	37.662	7.143
11	MLN_MDSC	37.313	8.735
12	MLN_Treg	37.172	7.315
13	LLG_NK	35.897	6.733
14	Gln	35.258	9.017
15	Urea	34.405	8.525
16	SP_NK	33.190	6.745
17	Glu	29.673	6.408
18	BM_Treg	28.774	5.156
19	Arg	28.193	6.427
20	SP_MDSC	28.090	6.399
21	His	27.378	5.305
22	Amm	27.090	5.504
23	Pea	26.350	4.946
24	LLG_Treg	24.550	5.240
25	MLN_CD8+ T eff	24.501	5.598
26	Cys	23.905	6.260
27	Ser	22.505	4.059
28	Gly	22.475	5.487
29	Taur	20.184	3.423
30	Val	18.793	3.295
31	Phe	18.248	3.316
32	SP_Treg	18.174	2.928
33	Tyr	18.059	3.307
34	Asn	17.938	3.286
35	Lys	16.521	3.447
36	Ala	15.381	2.984
37	Leu	15.131	2.585
38	Pro	15.086	2.980
39	Thr	14.927	2.777
40	Met	14.816	2.671
**41**	**Ile**	**14.180**	**2.638**
**Mean AUC=0.923 for 41 features**			

B. RF, initial model, RFE

Iteration	# of features	Mean AUC	Remove lowest ranked feature
**1**	**41**	**0.923**	**Ile**
2	40	0.932	Thr
3	39	0.932	Pro
4	38	0.941	Ala
5	37	0.949	Met
6	36	0.934	Leu
7	35	0.936	Lys
8	34	0.935	Phe
9	33	0.946	SP_Treg
10	32	0.945	Tyr
11	31	0.949	Asn
12	30	0.949	Val
13	29	0.948	Taur
14	28	0.933	LLG_Treg
15	27	0.943	Gly
16	26	0.942	Ser
17	25	0.933	Cys
18	24	0.940	MLN_CD8+ T eff
19	23	0.945	Pea
20	22	0.939	SP_MDSC
21	21	0.953	Amm
22	20	0.943	BM_Treg
23	19	0.943	Arg
**24**	**18**	**0.955**	**Glu**
25	17	0.945	His
26	16	0.944	Gln
27	15	0.941	SP_NK
28	14	0.945	MLN_Treg
29	13	0.951	LLG_NK
30	12	0.942	MLN_MDSC
31	11	0.930	Urea
32	10	0.925	Orn
33	9	0.932	BM_CD8+ T eff
34	8	0.929	Citr
35	7	0.941	BM_MDSC
36	6	0.925	SP_CD8+ T eff
37	5	0.914	MLN_NK
38	4	0.867	BM_NK
39	3	0.854	LLG_MDSC
40	2	0.788	LLG_CD8+ T eff
41	1	0.718	Phser

C. RF, reduced model, feature list

Rank	Feature
**1**	**Phser**
**2**	**LLG_CD8+ T eff**
**3**	**LLG_MDSC**
**4**	**BM_NK**
**5**	**MLN_NK**
**6**	**SP_CD8+ T eff**
**7**	**BM_MDSC**
**8**	**Citr**
**9**	**BM_CD8+ T eff**
**10**	**Orn**
**11**	**Urea**
**12**	**MLN_MDSC**
**13**	**LLG_NK**
**14**	**MLN_Treg**
**15**	**SP_NK**
**16**	**Gln**
**17**	**His**
**18**	**Glu**
**Mean AUC=0.955 for 18 features**	

Performance for the reduced model across all algorithms remained strong (Table S17). Gradient Boost achieving an AUC of 0.955 ± 0.030 and Accuracy of 0.896 ± 0.044, XGBoost achieving an AUC of 0.952 ± 0.041 and Accuracy of 0.901 ± 0.044, Logistic Regression achieving an AUC of 0.917 ± 0.055 and Accuracy of 0.854 ± 0.050, and Random Forest achieving an AUC of 0.955 ± 0.032 and Accuracy of 0.902 ± 0.038. These values are comparable to the corresponding full models, indicating that compact feature sets preserve most discriminatory capacity for cancer versus no cancer. Notably, for XGBoost the optimal AUC occurred near the full feature set (38 of 41 features; Table S17), consistent with this algorithm distributing predictive signal across many correlated predictors.

Table S17. IMPACT Cancer vs No Cancer, Reduced Model Performance (top AUC features), related to Figure 3								
**Algorithm**	**# Features**	**AUC**	**Accuracy**	**Sensitivity**	**Specificity**	**Precision**	**F1 Score**	**Threshold**
**Gradient Boost**	10	0.955 (±0.030)	0.896 (±0.044)	0.955 (±0.036)	0.724 (±0.159)	0.913 (±0.046)	0.932 (±0.028)	0.48
**XGBoost**	38	0.952 (±0.041)	0.901 (±0.044)	0.953 (±0.041)	0.749 (±0.114)	0.918 (±0.036)	0.935 (±0.029)	0.64
**Logistic Regression**	24	0.917 (±0.055)	0.854 (±0.050)	0.949 (±0.045)	0.576 (±0.176)	0.870 (±0.048)	0.906 (±0.031)	0.11
**Random Forest**	18	0.955 (±0.032)	0.902 (±0.038)	0.940 (±0.040)	0.791 (±0.131)	0.931 (±0.040)	0.934 (±0.024)	0.62
"±" represents Standard Deviation of the performance metrics calculated across 30 random resampling iterations. Threshold represents the classification probability cutoff optimized via grid search to maximize the F1 score: Cancer is the positive class.								

To compare the biological signals captured by interaction aware IMPACT with those identified by conventional univariate mean difference testing, we visualized both outputs for cancer detection. Figure S2A summarizes mean differences for Cancer (CS+CR) versus No Cancer (1XPBS + no injections) across the 32 significant systemic immunometabolic features (Table S9; see also Figure 3C-F for directionality). Figure S2B summarizes the 18 features selected by IMPACT using RFE from the best performing Random Forest model (highest AUC; Table S16), with arrow directions mapped to the Cancer-associated direction shown in Figure 3C-F. Several patterns emerge.

First, mean difference testing highlights a broader "metabolite dominant" cancer signal, whereas IMPACT/RFE prioritizes a more "immune dominant" cancer signal, especially features that remain informative across the heterogeneity within the pooled Cancer group (CS+CR). This divergence is expected because the two approaches answer different questions: mean differences identify markers that shift on average (marginal effects), while IMPACT identifies markers that improve classification conditional on other features (multivariate contribution in the presence of correlations and interactions). In classifying Cancer, this distinction is amplified by the fact that "Cancer" intentionally pools two biological subtypes (CS and CR), meaning some metabolites can be subgroup dependent (strong in CS but weak or even reversed in CR). For example, mean difference plots show histidine rising in CS vs No Cancer, while the dominant CR-associated shifts emphasize other metabolites (e.g., ammonia/glutamate/phosphoserine), making a single "Cancer direction" for some amino acids harder to interpret from mean differences alone. In that setting, RFE naturally favors features that are (1) consistently cancer informative across CS and CR and (2) add nonredundant information such as LLG_MDSC and phosphoserine, which repeatedly emerge as top ranked features across models while potentially down weighting amino acids whose signal is diluted by pooling or is redundant with other correlated metabolites. Conceptually, this is why a metabolite such as phenylalanine can appear "statistically shifted" in a univariate view but still fail to survive multivariate pruning: its marginal shift may not translate into incremental discrimination once immune composition and other correlated amino acids are considered.

Second, a smaller subset of metabolites shows a directionally consistent with cancer in both CS and CR groups when each is contrasted against the No Cancer baseline. This is notable because it avoids the interpretability problem highlighted in Pattern 1, i.e., features whose "cancer direction" flips depending on whether the comparison is CS vs No Cancer or CR vs No Cancer. In other words, for these metabolites the signal behaves more like a shared Cancer main effect than a subgroup dependent interaction, strengthening the case that they reflect a systemic phenotype common to cancer bearing animals rather than subgroup specific biology or sampling variation. Specifically, ornithine, glutamate, phosphoserine, and phosphoethanolamine are increased in both CS and CR relative to No Cancer, while urea and citrulline are decreased in both CS and CR relative to No Cancer. That the same directionality holds across CS and CR suggests these features are comparatively robust to how the "Cancer" label is instantiated (CS vs CR) and

therefore are less likely to be artifacts of a particular subgroup contrast. Importantly, IMPACT largely recapitulates this cross subgroup consistency: among these concordant metabolites, it retains ornithine, glutamate, phosphoserine, urea, and citrulline, providing an orthogonal confirmation that these signals are not only statistically shifted in aggregate but also useful for classification within the multivariate, interaction-aware modeling framework. The main exception is phosphoethanolamine, which despite its consistent directionality by mean differences was not selected by RFE in the best performing Random Forest model. This discrepancy is not necessarily contradictory: feature elimination in a nonlinear classifier tends to prioritize predictive sufficiency over biological completeness, so a metabolite can show a reproducible group shift yet be dropped if it is (1) strongly correlated with another retained feature, (2) adds limited incremental discriminative power once immune cell and other metabolic features are included, or (3) is less stable across folds/bootstraps even if its mean difference is significant. Practically, this “concordant-but-not-selected” status makes phosphoethanolamine a reasonable candidate for targeted follow-up (e.g., correlation structure, stability selection, SHAP/feature attribution), rather than an automatic false positive.

Third, the intersection of (1) metabolites significant by mean difference testing and (2) metabolites prioritized by IMPACT is not randomly distributed across amino acid space. Instead, the shared features with concordant CS/CR directionality appear to concentrate around nitrogen handling and urea cycle adjacent metabolism, including ornithine, citrulline, urea, and glutamate. This clustering is informative because it suggests that the overlap between “statistically altered” and “predictive for classification” features is capturing a more pathway-coherent signal, rather than a broad, diffuse set of amino acid changes that may each reach significance but do not collectively resolve into a stable mechanistic theme. Biochemically, these metabolites sit at key junctions of systemic nitrogen balance: urea is the end-product of nitrogen disposal; ornithine and citrulline are canonical intermediates associated with arginine/ornithine flux and urea-cycle chemistry; and glutamate serves as a central nitrogen hub linking transamination and amino acid interconversion. The directional pattern observed here, higher ornithine and glutamate alongside lower citrulline and urea in both cancer subgroups, is therefore consistent with a coordinated reshaping of how nitrogen is processed, transported, or partitioned at the organismal level in cancer-bearing animals. While the present analysis does not by itself specify the causal drivers (e.g., tumor demand, host response, immune-metabolic remodeling, or organ-level changes in nitrogen disposal), the fact that these metabolites are (1) reproducibly shifted across CS and CR, (2) highlighted by univariate testing, and (3) retained by a multivariate classifier suggests they represent a robust, classification-relevant axis of systemic immunometabolic reprogramming. Analytically, this helps reconcile why mean differences can yield a comparatively large list of “altered amino acids” whereas IMPACT returns a smaller set: univariate tests are sensitive to any consistent shift, including multiple partially redundant metabolites within the same biochemical neighborhood, whereas model based selection tends to keep a minimal subset that best captures a discriminative trajectory. In this light, the shared urea cycle adjacent metabolites can be interpreted as a compact, pathway level signature that is both stable across cancer subtypes (CS and CR) and informative for cancer detection, making it especially attractive for downstream validation. Concretely, this observation motivates follow up testing (e.g., ratio features such as ornithine/citrulline or glutamate/urea; pathway enrichment/network mapping; and sensitivity analyses evaluating whether this “nitrogen axis” remains predictive when immune cell features are held constant), which would strengthen the mechanistic narrative without relying solely on individual metabolite p-values.

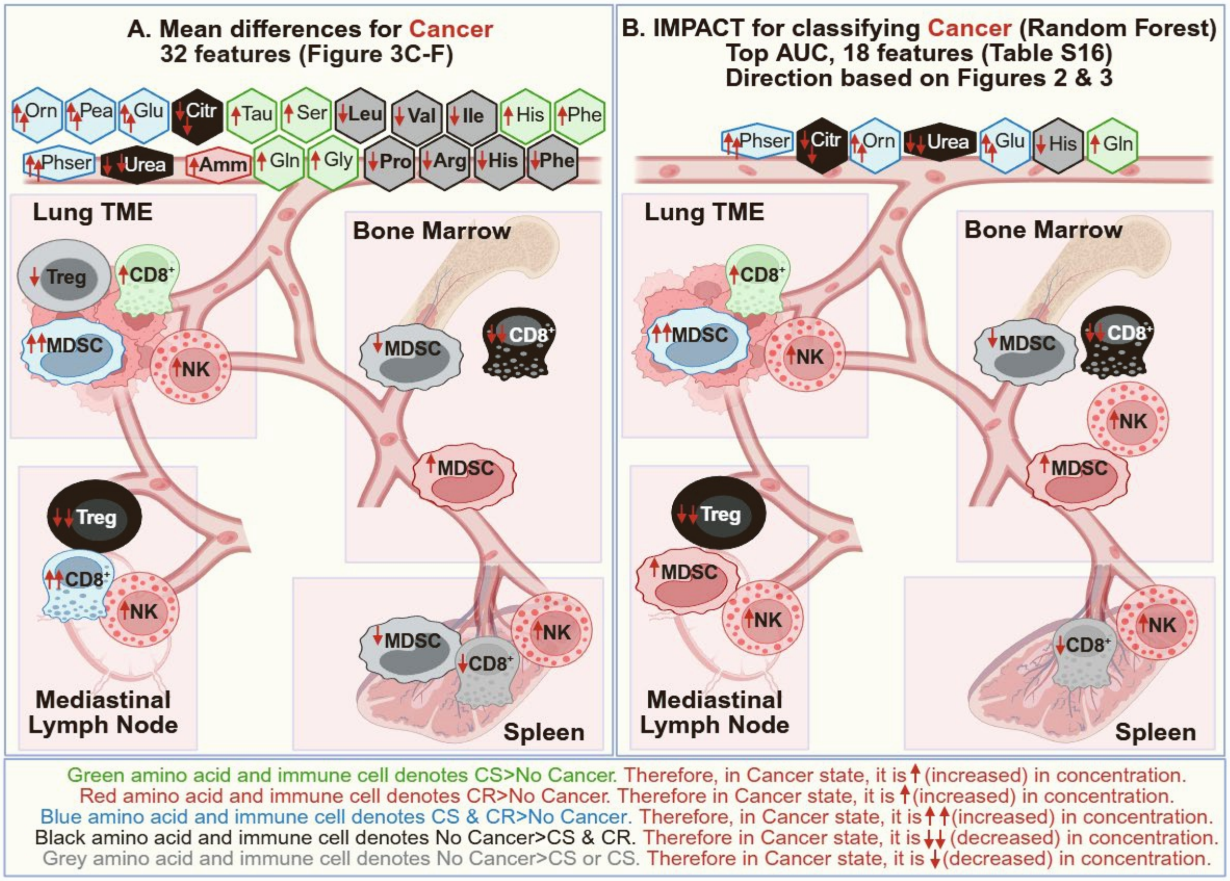

Figure S2. Systemic immunometabolic features comparing mean differences versus IMPACT to detect cancer, related to Figure 3.

(A) Mean difference profile for Cancer (CS+CR) versus No Cancer (1XPBS + no injections; Table S9) across 32 significant systemic immunometabolic features. Arrows indicate the direction of change in Cancer relative to No Cancer.

(B) IMPACT feature prioritization for cancer classification using recursive feature elimination (RFE). The Random Forest model with the best AUC selected 18 features (Table S16). For comparison with panel A, arrow directionality reflects the Cancer associated direction for each feature as shown in Figure 3C-F.

3. Data S3/Methods S3: QUANTIFICATION AND STATISTICAL ANALYSIS

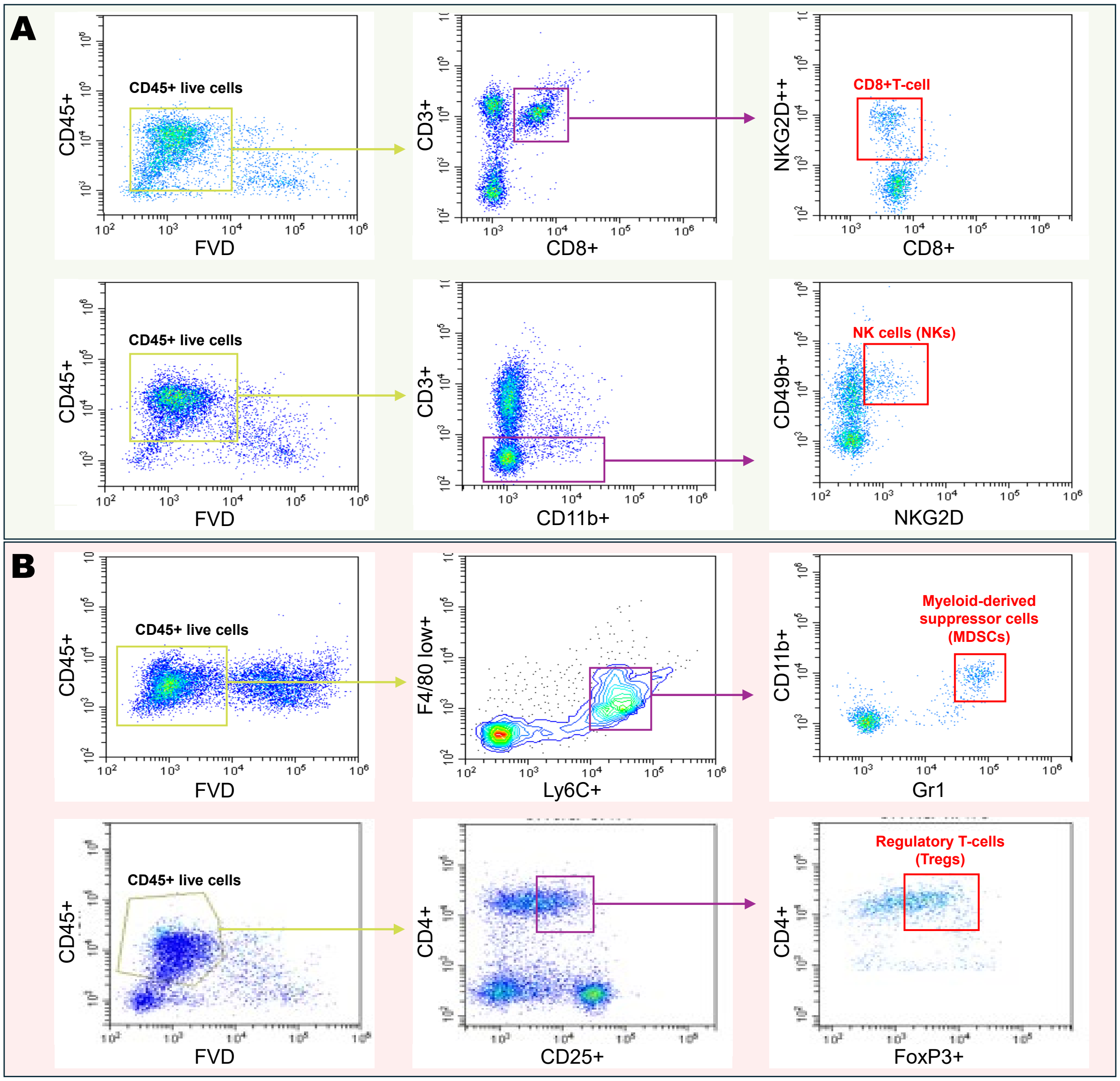

Figure S3. Flow cytometry gating strategy for identifying CD8^{+} T cells, NK cells, MDSCs, and Treg populations.

(A) Gating of CD8^{+} T cells and NK cells. Live leukocytes were identified by exclusion of fixable viability dye (FVD)-positive events and gating on CD45^{+} cells. CD3^{+}CD8^{+} cells were gated as CD8^{+} T cells (top row). NK cells were identified as CD3^{-}CD49b^{+}NKG2D^{+} events (bottom row). Representative plots show sequential gating from live CD45^{+} cells to lineage-specific subsets.

(B) Gating of myeloid-derived suppressor cells (MDSCs) and regulatory T cells (Tregs). For MDSCs (top row), CD45^{+} live cells were gated on F4/80low Ly6C^{+}, followed by CD11b^{+}Gr-1^{+} events to identify total MDSCs. For Tregs (bottom row), CD45^{+} live cells were gated on CD3^{+}CD4^{+} T cells, followed by CD25^{+}FoxP3^{+} events to define regulatory T cell populations. Representative plots illustrate each sequential gate used to isolate MDSCs and Tregs from lung, spleen, MLN, or bone marrow samples.

Table S18. Preprocessing data	
**File Name**	**Brief description of contents**
mydat_init.csv	Initial merged dataset prior to preprocessing (raw systemic immunometabolic features).
mydat_full.csv	Analysis dataset after basic cleaning/derivations but before imputation (all features and labels).
mydat_imputed.csv	Final modeling dataset with missing values (1.43%) imputed with missForest and ready for model training.

Table S19. Comparison of RFE vs SHAP								
**CS vs. CR, related to Figure 2**								
**Step 1, Initial Model 41 features**					**Step 2, RFE best AUC's # of features (Table S4C-S7C)**			
Method	Spearman	Top 10 overlap	Top 15 overlap	Top 20 overlap	Method	RFE best AUC's # features	Spearman	Top 10 overlap
GB	0.951	9/10	13/15	17/20	GB	12	0.685	9/10
XGB	0.877	9/10	12/15	17/20	XGB	22	0.842	9/10
LR	0.657	4/10	10/15	15/20	LR	21	0.743	8/10
RF	0.955	9/10	13/15	19/20	RF	21	0.945	9/10
**Cancer vs. No Cancer, related to Figure 3**								
**Step 1, Initial Model 41 features**					**Step 2, RFE best AUC # of features (Table S13C-S16C)**			
Method	Spearman	Top 10 overlap	Top 15 overlap	Top 20 overlap	Method	RFE best AUC's # features	Spearman	Top 10 overlap
GB	0.975	9/10	15/15	19/20	GB	10	0.794	10/10
XGB	0.929	8/10	12/15	18/20	XGB	38	0.929	7/10
LR	0.762	8/10	10/15	14/20	LR	24	0.763	8/10
RF	0.975	8/10	15/15	18/20	RF	18	0.754	8/10

In summary, this supplement supports the robustness and biological coherence of the IMPACT framework. First by comparing univariate mean difference profiles with multivariate feature prioritization, these results demonstrate that cisplatin sensitivity and cancer status are encoded as *coordinated* systemic shifts rather than isolated marker fluctuations. Second, the IMPACT framework is complementary to mean difference profiles, effectively reducing high dimensional biological complexity into stable, predictive signatures suitable for downstream mechanistic validation and translational assay design.
